# Homologous targeting nanoparticles for enhanced PDT against osteosarcoma HOS cells and the related molecular mechanisms

**DOI:** 10.1186/s12951-021-01201-y

**Published:** 2022-02-17

**Authors:** Yang Wang, Liang Zhang, Guosheng Zhao, Yuan Zhang, Fangbiao Zhan, Zhiyu Chen, Tao He, Yang Cao, Lan Hao, Zhigang Wang, Zhengxue Quan, Yunsheng Ou

**Affiliations:** 1 Department of Orthopedic Surgery, The First Affiliated Hospital of Chongqing Medical University, Chongqing, 400016 People’s Republic of China; 2 Department of Ultrasound, The First Affiliated Hospital of Chongqing Medical University, Chongqing, 400016 China; 3 Department of Orthopedic Surgery, The Second Affiliated Hospital of Chongqing Medical University, Chongqing, 400016 People’s Republic of China; 4 Department of Orthopedic Surgery, Children’s Hospital of Chongqing Medical University, Ministry of Education Key Laboratory of Child Development and Disorders, Key Laboratory of Pediatrics in Chongqing, China International Science and Technology Cooperation Base of Child Development and Critical Disorders, Chongqing, 400014 People’s Republic of China; 5 Department of Ultrasound Imaging, Second Affiliated Hospital of Chongqing Medical University, Chongqing, 400014 People’s Republic of China

**Keywords:** PDT, OS, Homologous targeting, Apoptosis, Ferroptosis

## Abstract

**Background:**

No prominent advancements in osteosarcoma (OS) treatment have been made in the past 20 years. Although photodynamic therapy (PDT) is an emerging technique for cancer therapy, the lack of targeted photosensitizers for OS treatment severely limits its applications.

**Results:**

In this study, we constructed a potential theranostic nanoplatform by using (poly (lactic-co-glycolic) acid (PLGA) nanoparticles (NPs) encapsulating IR780 into the shell (PLGA-IR780 NPs), which were further camouflaged with human OS cell membranes from the HOS cell line (MH-PLGA-IR780 NPs). These constructed NPs showed the capacity for homologous targeting with excellent photoacoustic (PA)/fluorescence (FL) imaging ability. Benefitting from their homologous targeting capacity, MH-PLGA-IR780 NPs obviously promoted cell endocytosis in vitro and tumor accumulation in vivo, which could further improve PDT performance under near-infrared (NIR) irradiation. In addition, to their homologous targeting and PA/FL dual-mode imaging ability, MH-PLGA-IR780 NPs had advantages in penetrating deeper into tumor tissues and in real-time dynamic distribution monitoring in vivo, which laid a foundation for further clinical applications in OS. Moreover, we demonstrated that PDT guided by the constructed NPs could significantly induce HOS cells apoptosis and ferroptosis via excessive accumulation of reactive oxygen species (ROS), and further determined that the potential anticancer molecular mechanism of apoptosis was triggered by the release of cytochrome c-activated mitochondrial apoptosis (endogenous apoptosis), and that ferroptosis caused the activation of nuclear receptor coactivator 4 (NCOA4)-mediated ferritinophagy and the inactivation of glutathione peroxidase 4 (GPX4), synergistically leading to excessive accumulation of Lipid-ROS and Lipid peroxides (LPOs). Concurrently, MH-PLGA-IR780 NPs-guided PDT also showed an obvious inhibitory effect on tumor growth in vivo.

**Conclusion:**

These results suggest that this homologous targeting-based theranostic nanoplatform provides an effective method to improve PDT performance in OS and contributes a new and promising approach for OS therapy.

**Graphical Abstract:**

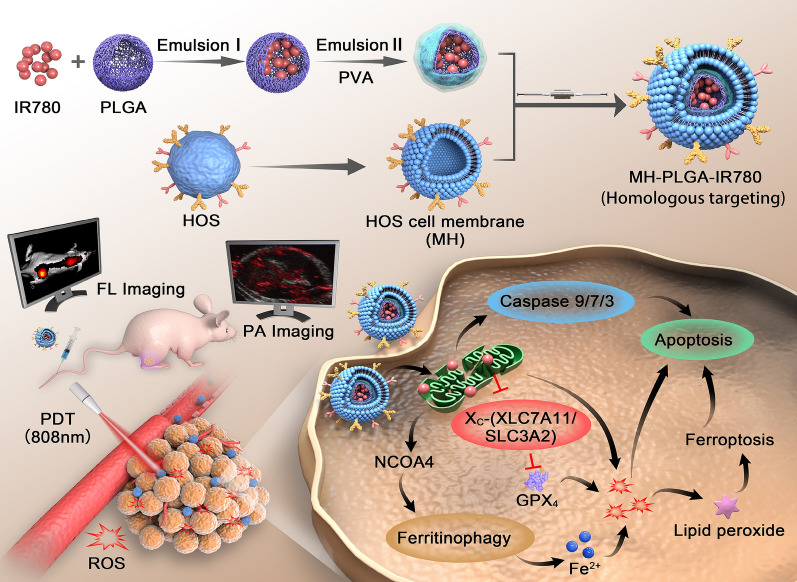

**Supplementary Information:**

The online version contains supplementary material available at 10.1186/s12951-021-01201-y.

## Background

Surgery and neoadjuvant chemotherapy are the main clinical treatments for osteosarcoma (OS), but the 5-year survival rate of patients with OS remains between 65% and 70% [1]. As a potential anticancer therapeutic method, photodynamic therapy (PDT) induces cell death depending on excessive accumulation of cytotoxic reactive oxygen species (ROS) [2, 3]. However, the poor targeting of photosensitizers (PSs) to OS cells seriously limits PDT performance and further clinical application [4]. Therefore, overcoming the poor targeting of PSs for OS treatment should be considered an advanced strategy to improve the innate characteristics of PDT.

With the rapid development of nanotechnology, utilizing cancer cell membranes for nanoparticle surface functionalization presents a potential targeting method [5–7]. Relative to the accumulation of traditional nanoparticles at tumor sites via the enhanced permeation and retention (EPR) effect [8], as a top-down approach, cancer cell membrane coated nanoparticles (CCMCNPs), featuring a tight core–shell structure with a “right-side-out” membrane topological manner orientation [6, 9], have been demonstrated to provide the capability to target tumors by their homologous targeting property and immune escape ability [9, 10]. This biomimetic engineering strategy of coating NPs with cell membranes could be successfully implemented due to its unique advantage of completely replicating the surface antigenic diversity of the source cells on the engineered NPs. Therefore, based on this emerging method, poly(lactic-co-glycolic) acid (PLGA) NPs were camouflaged with human OS cell membranes from the HOS cell line (membrane-HOS-PLGA, MH-PLGA), with an aim to synthesize the homologous targeting NPs to improve cell endocytosis in vitro and tumor accumulation in vivo.

To further exploit the multifunctional homologous targeting-based nanoplatform with imaging PDT functions, IR780 was selected for encapsulation into the constructed homologous targeting-based nanoplatform (MH-PLGA-IR780 NPs). As a heptamethine cyanine molecule, IR780 has been used as a probe for photoacoustic (PA)/fluorescence (FL) imaging of tumors for accurate diagnosis and is activated under near-infrared (NIR) irradiation (808 nm), and its unique light-excited nanoplatform has been used as a promising nanoagent for some tumor therapies [11–14]. Benefitting from their homologous targeting and NIR FL/PA dual-mode imaging capabilities, these NPs have been verified to penetrate deeper into tumor tissues and to monitor the real-time dynamic distribution in vivo [15, 16].

**Scheme 1:**
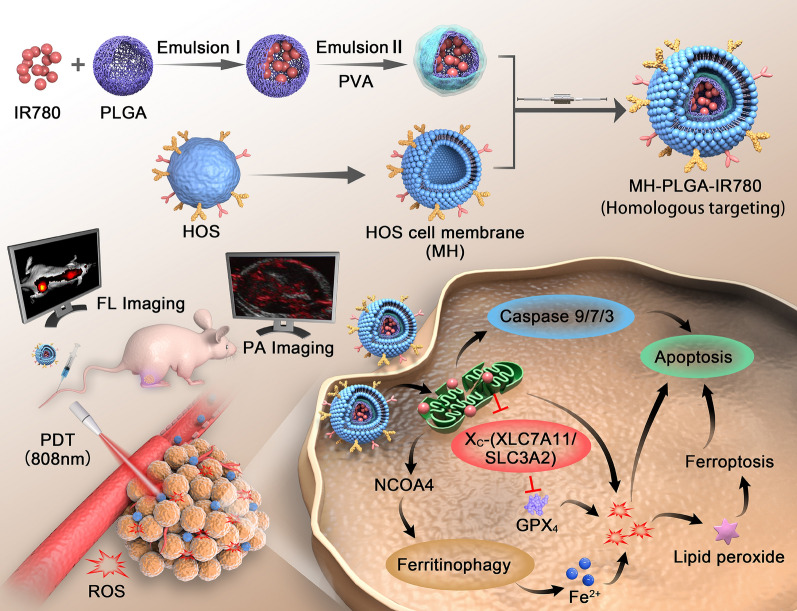
Schematic illustration of the construction of the MH-PLGA-IR780 NPs and the specific killing mechanism of the targeted theranostic nanoplatform-mediated PDT approach

Although homologous targeting/imaging nanoplatforms have been reported to guide PDT in other cancers, there are few similar reports in OS. In addition, the specific cytotoxic mechanism of homologous targeting-mediated PDT is unclear. Currently, the main death mode of singlet oxygen-mediated PDT against solid tumors has long been considered to be apoptosis [17–19]. In our engineered theranostic nanoplatform-mediated PDT research, surprisingly and excitingly, we were more intrigued by the significant cytotoxic effect mediated through the induction of ferroptosis. Unlike apoptosis, ferroptosis is dependent on the overproduction of tailored lipid peroxides (LPOs), excessive accumulation of the cellular labile iron pool (LIP) and defective lipid peroxidation repair via inhibition of glutathione peroxidase 4 (GPX4) [20]. Due to tumor heterogeneity, the phenotypic diversity of tumor cells and activation of antiapoptotic pathways leading to tumor resistance to apoptosis, PDT cannot effectively eradicate tumors via a single mode of cell death [21, 22]. In addition, the excessive accumulation of redox-active ions is helpful for improving PDT performance via accumulation of hydroxyl radicals (·OH), another ROS through the Fenton reaction [23, 24]. In the present study, our results demonstrated that targeted PDT exerted a significant cytotoxic effect via synergistic induction of apoptosis and ferroptosis in vitro and markedly inhibited tumor growth in vivo. Moreover, the possible molecular cell death pathway of combined apoptosis and ferroptosis underlying the effectiveness of this homologous targeting-based theranostic nanoplatform-mediated PDT strategy is discussed (Scheme 1).

### Reagents and methods

#### Reagents

Biocompatible PLGA (50:50, MW: 12,000 Da) was obtained from Ruixi Biotech Co., Ltd. (Xian, China). IR780 iodide, G418 and B27 were purchased from Sigma-Aldrich (St. Louis, MO, USA). Dichloromethane (CH_2_Cl_2_) was purchased from Beijing Bailingwei Technology Corp. (China). MitoTracker™ Deep Red FM (MitoTracker) and C-11 BODIPY™ 581/591 (C11-BODIPY) were purchased from Invitrogen (Carlsbad, CA, USA). Fetal bovine serum (FBS) and Dulbecco’s-modified Eagle’s medium (DMEM) were purchased from HyClone (Logan, UT, USA). The cell viability and cytotoxicity assay kits, namely, Cell Counting Kit-8 (CCK-8), Liperfluo and FerroOrange, were purchased from Dojindo Molecular Technologies (Kumamoto, Japan). The annexin V-FITC/propidium iodide (PI) double-staining test kit was purchased from KeyGen Biotech (Nanjing, China). RIPA lysis buffer, phenylmethanesulfonyl fluoride (PMSF), a bicinchoninic acid (BCA) protein assay kit, a Membrane and Cytosol Protein Extraction Kit (cat. P0033), bovine serum albumin (BSA), 1,1’-dioctadecyl-3,3,3’,3’-tetramethylindocarbocyanine perchlorate (DiI), a JC-1 mitochondrial membrane potential (MMP) assay kit (cat. C2006), a Membrane and Cytosol Protein Extraction Kit (cat. P0033), 2, 7-dichlorodihydrofluorescein-diacetate (DCFH-DA) (S0033S), N-acetyl-L-cysteine (NAC) (cat. S0077), an adenosine triphosphate (ATP) assay kit (cat. S0026), and a mouse anti-human GAPDH antibody (cat. AG019-1) were obtained from Beyotime Biotech (Shanghai, China). YF633-Phalloidin was sourced from Everbright, Inc. (US). Rabbit anti-human Bax (cat. D2E11), rabbit anti-human Bcl-2 (D55G8) (cat. 4223), rabbit anti-human cytochrome c (cat. 11940), rabbit anti-human cleaved caspase-7 (cat. 8438), rabbit anti-human cleaved caspase-9 (cat. 20750), and rabbit anti-human cleaved caspase-3 (cat. 9664) antibodies, and a ferroptosis Antibody Sampler Kit (cat. 29650) were purchased from Cell Signaling Technology (Boston, MA, USA). Z-VAD-FMK (cat. S7023), necrostatin-1 (Nec-1) (cat. S8037), bafilomycin A1 (Baf-A1) (cat. S1413), ferrostatin-1 (Fer-1) (cat. S7243) and deferoxamine (DFO) (cat. S5742) were sourced from Selleck (Houston, USA). Antibodies specific for PCNA (cat. 10205-2-AP) and Ki67 (cat. 27309-1-AP) were obtained from Proteintech (USA). Epidermal growth factor (EGF), and basic fibroblast growth factor (bFGF) were obtained from PeproTech (USA).

### Cell culture

The following cell lines were purchased from the American Type Culture Collection (ATCC, Manassas, VA, USA): the human OS cell lines HOS, MG63, and 143b; the murine OS cell line K7M2; the murine breast cancer cell line 4T1; and human non-small-cell lung cancer cells line A549. These cells were maintained in DMEM supplemented with 10% FBS, 100 μg/mL penicillin, and 100 μg/mL streptomycin. Cells were maintained in a humidified atmosphere with 5% CO_2_ at 37 °C. The human fetal osteoblastic cell line HFOB 1.19 was also obtained from the ATCC, and the cells were cultured in Dulbecco's modified Eagle’s medium F-12 nutrient mixture (DMEM/F12) supplemented with 2.5 mM l-glutamine and 0.3 mg/mL G418 in addition to 10% FBS, 100 μg/mL penicillin, and 100 μg/mL streptomycin. HFOB 1.19 cells were maintained in a humidified 5% CO_2_ atmosphere at a permissive temperature of 34 °C.

### Synthesis of the MH-PLGA-IR780 NPs

(i) The water-in-oil-in-water (W/O/W) double-emulsion method was used to synthesize PLGA-IR780 NPs. Briefly, PLGA (50 mg) and IR780 (2 mg) were dissolved in CH_2_Cl_2_, and then primary emulsification was performed using an ultrasonic probe (Sonics & Materials, Inc., USA) (50 W, 3 min). Subsequently, 4% polyvinyl alcohol (PVA) (10 mL) was added to the emulsified solution, which was homogenized by a second sonication to form a W/O/W double emulsion (35 W, 3 min). Then, 2% isopropanol solution (10 mL) was added to the prepared emulsion, which was mechanically stirred for 2 h to remove CH_2_Cl_2_ followed by centrifugation (12,000 rpm for 6 min) to obtain PLGA-IR780 NPs. (ii) To prepare the HOS cell membranes, HOS cells were grown in T-75 culture flasks to full confluence, detached with 2 mM EDTA in phosphate-buffered saline (PBS), and washed three times in PBS by centrifugation at 800 rpm for 5 min. Subsequently, the cells were lysed with a Membrane and Cytosol Protein Extraction Kit A containing PMSF (100:1) at −80 °C with three repeated freeze–thaw cycles followed by centrifugation (800 rpm for 5 min) to collect the cellular supernatant. Then, the precipitate was obtained by centrifugation (12,000 rpm, 30 min) at 4 °C and resuspended in PBS. The above step was repeated three times. Finally, the precipitate was dispersed in PBS followed by centrifugation (3000 rpm, 30 min) at 4 °C, and the upper solution was the HOS cell membrane (MH). (iii) MH-PLGA-IR780 NPs were finally synthesized by physically extruding the mixture of PLGA-IR780 NPs (5 mg/mL, 0.5 mL) and MH (5 mg/mL, 0.5 mL) at the same concentration for 11 passes through a 400 nm polycarbonate porous membrane on a mini extruder (Avanti Polar Lipids, USA). The final product was stored at 4 °C for later use.

### Characterization of the MH-PLGA-IR780 NPs

The size distribution, and zeta-potentials of various NPs and their stability of the NPs in fetal blood serum (10%) and PBS over seven days were determined using dynamic light scattering (DLS, Malvern Instruments, UK). The morphologies and structures of the NPs were observed using transmission electron microscopy (TEM, Hitachi H-7600, Japan). The optical absorption of the different NPs and free IR780 was determined with an ultraviolet–visible (UV–vis) spectrophotometer (US-2550, Shimadzu, Japan). A standard concentration curve of free IR780 measured at a wavelength of 798 nm was constructed to calculate the amount of IR780 encapsulated into the MH-PLGA NPs. The encapsulation efficiency (EE) and encapsulation content (EC) of IR780 were calculated by Eqs. (1) and (2)

1 $${\text{EE}}_{{{\text{IR78}}0}} \left( \% \right) \, = \, \left( {{\text{mass of total IR78}}0 \, - {\text{ mass of unentrapped IR78}}0/{\text{mass of total IR78}}0} \right) \, \times {1}00\%$$

2 $${\text{EC}}_{{{\text{IR78}}0}} \left( \% \right) \, = \, \left( {{\text{mass of total IR78}}0 \, - {\text{ mass of unentrapped IR78}}0/{\text{mass of total PLGA NPs}}} \right) \, \times {1}00\%$$

### Cellular uptake and deep penetration capability of the MH-PLGA-IR780 NPs

Confocal laser scanning microscopy (CLSM; Nikon A1 + R, Japan) and flow cytometry (FC; BD FACSvantage SE, USA) were used to detect the cellular uptake of different NPs. Typically, HOS cells (1 × 10^5^/dish) were seeded into a confocal cell culture dish. After 24 h of incubation, the culture medium was replaced with serum-free medium containing PLGA NPs, PLGA-IR780 NPs or MH-PLGA-IR780 NPs (stained with DiI; λ excitation/λ emission = 549 nm/565 nm) for 1, 2, 3 or 4 h (IR780, 5 μg/mL). Then, the cells were fixed with 4% formaldehyde for 10 min and washed with PBS. After incubation for different time intervals with various NPs, nuclei were stained with DAPI (blue fluorescence; λ excitation/λ emission = 364 nm/454 nm). The fluorescence images were directly recorded by CLSM. Moreover, the quantitative cellular uptake of PLGA NPs, PLGA-IR780 NPs, and MH-PLGA-IR780 NPs at different time intervals was quantified and analyzed by FC.

The penetration of the MH-PLGA-IR780 NPs was estimated in vitro using 3D tumor spheroid models. HOS tumor spheres (1 × 10^5^/well) were cultured in 6-well ultralow attachment plates (Corning, Tewksbury, MA) in stem cell medium that consisted of serum-free DMEM/F12 supplemented with 20 ng/mL EGF, 20 ng/mL bFGF, and B27. The medium was changed every two days for ten days. Then, the medium was replaced with DiI-labelled NPs dispersed in DMEM/F12 (1 mL, 5 μg/mL). After 4 h of coincubation, the 3D tumor spheroids were stained with DAPI for 10 min and observed using CLSM.

### Homologous targeting and macrophage uptake assay

To verify the homologous targeting of the MH-PLGA-IR780 NPs, different kinds of cells (HOS, 4T1, A549, HFOB 1.19, K7M2, MG63 and 143b cells) (1 × 10^5^/dish) were seeded into a confocal cell culture dish for 24 h of incubation. Before the test, the growth medium was replaced with DiI-labelled MH-PLGA-IR780 NPs at a concentration of 5 μg/mL for 4 h of coincubation. Then, the cells were fixed with 4% formaldehyde for 10 min and washed with PBS. Next, the HOS cell nuclei were labelled with DAPI for 10 min. Finally, the intracellular uptake of the NPs was observed using CLSM and quantitatively analyzed by FC.

Next, to verify the immune escape ability of the MH-PLGA-IR780 NPs, RAW 264.7 cells (1 × 10^5^/dish) were seeded in a laser confocal cell culture dish. After a 24 h incubation, the growth medium was replaced with serum-free medium containing DiI-labeled NPs (5 μg/mL) for 4 h of coincubation. Subsequently, the cells were fixed with 4% formaldehyde for 10 min and washed with PBS. YF633-phalloidin (10 min) and DAPI (30 min) were used to label the cytoskeleton and nuclei, respectively, of the RAW 264.7 cells. The uptake of various NPs by macrophages was finally observed by CLSM.

### FL imaging/biodistribution and PA imaging of the MH-PLGA-IR780 NPs in vivo

HOS tumor-bearing mice were intravenously injected with suspensions of different NPs (PLGA NPs, PLGA-IR780 NPs, MH-PLGA NPs, MH-PLGA-IR780 NPs) suspension (5 mg/mL, in 200 μL). Subsequently, NIR FL images were collected preinjection and 1, 3, 6 and 24 h post injection, and the relative FL intensity of each tumor region was measured with IndiGo 2.0.5.0 (Berthold Technologies, Germany). Ex vivo imaging was performed on the major organs and tumor tissues 6 h post injection to detect the biological distribution of the aforementioned NPs.

A Vevo LAZR Photoacoustic Imaging System (VisualSonics Inc., Toronto, Canada) was used to determine the PA performance of the PLGA-IR780 NPs and MH-PLGA-IR780 NPs. It has been reported that the PA signal intensity of IR780 is highest at an activation wavelength of 800 nm [25]. HOS tumor-bearing mice were intravenously injected with the PLGA-IR780 NPs and MH-PLGA-IR780 NPs suspensions (5 mg/mL, in 200 μL). PA images were collected at different time points (pre-injection, 1, 2, 3, 6 and 24 h post-injection), and the corresponding PA signal intensities were measured with a Vevo LAZR System.

### Cell viability assay

A CCK-8 assay was used to determine cell viability after the different treatments. Briefly, HOS cells were seeded into 96-well plates at a density of 5,000 cells/well and incubated overnight for adherence. To determine the safety of the different NPs in vitro, HOS cells were incubated with various concentrations of NPs (NPs: 0.0, 0.2, 0.4, 0.6, 0.8, and 1.0 mg/mL; IR780: 0.0, 6.5, 13.0, 19.5, 26.0, and 32.5 μg/mL) for different lengths of time (0, 12, 24 and 48 h). To assess the change in cell viability after pretreatment with different inhibitors (z-VAD-FMK, Nec-1, Baf-A1, DFO, Fer-1, NAC), HOS cells were preincubated with the aforementioned inhibitors for 24 h before MH-PLGA-IR780 NP-guided PDT. Following the different treatments, 10 μL of CCK-8 reagent was added to each well and incubated for an additional 1 h. Finally, the plates with cells were placed in a microplate reader (MK3, Thermo Scientific) to measure the absorbance at 450 nm. Cell viability was calculated using the following equation: cell viability (%) = experimental group absorbance value/control group absorbance value × 100%.

### Measurement of intracellular ROS

Intracellular ROS were detected using DCFH-DA (λ excitation/λ emission = 488 nm/530 nm). Typically, HOS cells (1 × 10^5^/dish) were seeded into a laser confocal cell- culture dish. After a 24 h incubation, the medium was replaced with serum-free medium with or without NPs (PLGA-IR780 NPs or MH-PLGA-IR780 NPs (IR780: 5 μg/mL)) followed by incubation for another 4 h. Then, the cells in the laser group received irradiation with an 808 nm laser at a power density of 1.5 W/cm^2^ for 2 min. The cells were then incubated in serum-free medium containing 10 μM DCFH-DA in the dark at 37 °C for 30 min. Next, the cells were washed with PBS to remove excess DCFH-DA. Finally, the cells were immediately observed by CLSM to detect the intracellular ROS levels, treated with trypsin, collected in 200 µL PBS and detected by FC.

### MMP assay

The MMP (Δψm) was measured using a JC-1 assay kit. HOS cells (1 × 10^5^/dish) were seeded into a confocal cell culture dish for 24 h of incubation. After adherence, the HOS cells were divided into different treatment groups (control, laser alone, PLGA-IR780 NPs, laser + PLGA-IR780 NPs, MH-PLGA-IR780 NPs, and laser + MH-PLGA-IR780 NPs (IR780: 5 μg/mL). After intervention, all cells were cultured in fresh medium containing 1 mL of JC-1 staining solution (10 μM) and incubated for 30 min in the dark at 37 °C under 5% CO_2_. Then, the cells were washed with ice-cold JC-1 buffer, and JC-1 aggregates (λ excitation/λ emission = 585 nm/590 nm) and monomers (λ excitation/λ emission = 514 nm/529 nm) were observed by CLSM. To quantify Δψm, after exposure to different treatments, the cells were trypsinized, collected in medium containing JC-1 staining solution (10 μM), and incubated in the same environment for 30 min. After washing with ice-cold JC-1 buffer, the cells were suspended in 200 µL of PBS for FC analysis.

### TEM

TEM was used to detect the changes in mitochondrial morphology after different treatments to assess ferroptosis induction. HOS cells were seeded overnight into a 6-well plate at a density of 10^5^ cells/well to allow adherence and then divided into groups for various treatments (control, laser alone, PLGA-IR780 NPs, laser + PLGA-IR780 NPs, MH-PLGA-IR780 NPs, and laser + MH-PLGA-IR780 NPs (IR780: 5 μg/mL)). Next, the cells were trypsinized, collected, and fixed with 2.5% glutaraldehyde and 1% osmic acid. The cells were then dehydrated through an ethanol gradient and acetone, embedded, sliced, and stained with 3% uranyl acetate-lead citrate. Finally, the cells were examined by TEM (JEM-1400 Plus, JEOL, Japan).

### LPO and Lipid-ROS measurements

To determine the levels of LPOs and Lipid-ROS, HOS cells (1 × 10^5^/dish) were seeded into a confocal cell-culture dish for 24 h of incubation. Then, the cells were subjected to different treatments (control, laser alone, PLGA-IR780 NPs, laser + PLGA-IR780 NPs, MH-PLGA-IR780 NPs, laser + MH-PLGA-IR780 NPs (IR780: 5 μg/mL)). Subsequently, the cells were stained with 5 μM LPO (λ excitation /λ emission = 534 nm/535 nm) or 2 μM C11-BODIPY (λ excitation/λ emission = 500 nm/510 nm) for 30 min at 37 °C in the dark. Next, the cells were washed with PBS to remove the excess LPO and C11-BODIPY. Finally, CLSM was used to evaluate LPO and Lipid ROS levels in the cells, which were then trypsinized and collected for FC analysis.

### Fe^2+^ detection

FerroOrange (λ excitation/λ emission = 561 nm/570 nm) was used to determine the level of intracellular Fe^2+^. Briefly, HOS cells were seeded into a confocal cell culture dish at a density of 10^5^ cells/well overnight to allow adherence and then treated with laser alone, PLGA-IR780 NPs, laser + PLGA-IR780 NPs, MH-PLGA-IR780 NPs, or laser + MH-PLGA-IR780 NPs (IR780: 5 μg/mL). After 12 h of NIR treatment, the cells were stained with 1 μM FerroOrange for 30 min at 37 °C in the dark. Next, the cells were washed with PBS to remove the excess FerroOrange. Finally, the cells were observed by CLSM to detect the intracellular Fe^2+^ levels, treated with trypsin, collected in 200 µL of PBS and detected by FC.

### Cell apoptosis assay

Apoptosis was analyzed by FC using annexin V-FITC/PI staining. Briefly, cells were seeded into 6-well plates (1 × 10^5^ cells/well) and incubated overnight for adhesion. Then, the cells were exposed to laser alone, PLGA-IR780 NPs, laser + PLGA-IR780 NPs, MH-PLGA-IR780 NPs, or laser + MH-PLGA-IR780 NPs (IR780: 5 μg/mL). After the treatments, the cells were collected, washed twice with ice-cold PBS, and stained with annexin V-FITC and PI according to the manufacturer’s instructions. Finally, the samples were suspended in 200 µL of PBS and then analyzed by FC.

### Western blot analysis

After HOS cells were treated according to the different regimens, they were lysed with RIPA lysis buffer containing PMSF and phosphatase inhibitor within a specified period of time to extract the total protein in the cells. Protein samples (30 to 50 μg/lane) were separated on a 10–12% gel by SDS-PAGE and transferred to polyvinylidene fluoride (PVDF) membranes, which were blocked with 5% skim milk for 1.5 h at room temperature and then incubated overnight with the corresponding primary antibody (1:1000) at 4 °C. Next, the membranes were washed with Tris-buffered saline with Tween 20 (TBST) and incubated with the secondary antibody (1:8000) at 37 °C for 1 h. Finally, the immunoreactive protein bands on the membrane were detected with an enhanced chemiluminescence (ECL) detection system and developed on film.

### Intracellular ATP level

An ATP assay kit was used to determine the intracellular ATP level. Briefly, HOS cells were grown in 6-well plates at a density of 10^5^ cells/well and incubated overnight for adhesion. After the cells were exposed to laser alone, PLGA-IR780 NPs, laser + PLGA-IR780 NPs, MH-PLGA-IR780 NPs, and laser + MH-PLGA-IR780 NPs (IR780: 5 μg/mL), the cells were trypsinized and centrifuged (12,000 rpm for 5 min) at 4 °C. Finally, the cell supernatant was collected for RLU detection with a luminometer equipped with a multimode reader (260-Bio, Thermo Fisher Scientific, USA).

### Xenograft tumor model

Thirty male BALB/c nude mice (4 weeks old) were supplied by the Experimental Animal Center of Chongqing Medical University. All animal studies were approved by the Ethics Committee of Chongqing Medical University. The mice were housed with free access to a commercial diet and water under specific pathogen-free conditions. After the mice were acclimated for 1 week prior to initiation of the study, HOS tumor-bearing mice were established by subcutaneous injection of 200 μL of sterile PBS containing a HOS cell suspension at a density of 10^6^ cells/mL. After the tumor volumes reached 50 mm^3^, different treatments were initiated. The thirty mice were randomized into the following 6 groups: (1) control, (2) laser only, (3) PLGA-IR780 NPs, (4) laser + PLGA-IR780 NPs, (5) MH-PLGA-IR780 NPs, and (6) laser + MH-PLGA-IR780 NPs (laser power: 2 W/cm^2^, 5 min of irradiation) (PLGA concentration: 5 mg/mL, volume of 200 µL). The tumor sizes and mouse weights were measured every 4 days for 16 days after treatment, with tumor volumes calculated according to the following formula: 1/2 × a^2^b (where a is the short axis and b is the long axis of the tumor). Mice were sacrificed under anesthesia on day 16, and the xenograft tumors of each animal were weighed and analyzed.

To avoid the photothermal effect (PTT) and to ensure that any effects were due only to PDT, the temperature of the tumor region was monitored during irradiation with a Xenogen IVIS Spectrum imaging system (PerkinElmer, USA) so that remained below 42 °C. In vitro assays were performed on ice.

### Hematoxylin–eosin (H&E) and immunohistochemistry (IHC)

After sacrifice, tissues from the xenograft tumors, hearts, livers, spleens, kidneys, and lungs of mice were harvested and fixed with 10% formalin for histopathological studies. After fixation, the tissues were dehydrated in an ethanol gradient and xylene, embedded in paraffin, sliced into sections, and stained with H&E. The expression of PCNA and Ki67 in xenograft tumor tissues was detected by IHC. Briefly, the paraffin-embedded specimens were separated, fixed with 4% paraformaldehyde, and embedded in paraffin. After embedding, the specimens were discontinuously cut into 4-mm-thick sections with a microslicer. Tumor sections were blocked and immunostained with antibodies targeting Ki67 (1:200) or PCNA (1:200). Finally, images were acquired using a microscope, and PCNA and Ki67 expression was evaluated by counting the number of positive cells from 5 randomly selected fields in the residual viable tumor tissue among the necrotic areas under a light microscope at a magnification of 200× . Data are presented as the percentages of positive cells.

### Biosafety of the MH-PLGA-IR780 NPs and PDT

To determine the toxicity of the MH-PLGA-IR780 NPs, they were injected into BALB/c nude mice (PLGA concentration: 5 mg/mL, volume: 200 µL) that had been randomly divided into 5 groups. Twenty-five mice were euthanized on days 0, 1, 7, 14, and 28 post injection. The vital organs were collected for H&E staining, and blood samples were sent for blood index (routine blood and biochemistry) analyses. In addition, the methods for determining the biosafety of MH-PLGA-IR780 NPs-mediated PDT and the other parallel groups were consistent with the aforementioned methods and were performed after the mice were sacrificed at 16 days post irradiation.

### Statistical analysis

All data are expressed as the mean ± SD values and were analyzed with SPSS 22.0 software. Single Student’s t-test and one-way ANOVA were used to determine statistical significance between pairs of groups and three or more groups, respectively. Significance levels are shown as *p < 0.05, **p < 0.01, and ***p < 0.001.

## Results

### Preparation and characterization of MH-PLGA-IR780 NPs

MH-PLGA-IR780 NPs, a homologous targeting NPs with a shell/core structure (MH outside the shell and IR780 inside the shell) were prepared using a double-emulsion approach and the physical extrusion method. The engineered NPs achieved tumor targeting to the HOS cell line and had superior FL/PA imaging performance in vivo. Under NIR irradiation (808 nm), targeted PDT had the potential to cause mitochondrial dysfunction and led to the excessive accumulation of ROS, LPOs, and Lipid-ROS. Apoptosis and ferroptosis acted as mean death modes of the constructed targeting NPs-mediated PDT. The specific molecular pathway of apoptosis was revealed by the release of cytochrome c-activated mitochondrial apoptosis, and the activation of NCOA4-mediated ferritinophagy and the passivation of GPX4 synergistically induced another RCD, ferroptosis. More importantly, the relationship between cell apoptosis and ferroptosis induced by this targeted theranostic nanoplatform-mediated PDT was verified by the pretreatment with ferroptosis inhibitors (DFO and Fer-1), and it was finally demonstrated that ferroptosis acted as a “death switch” in this targeted PDT (Scheme 1).

**Fig. 1:**
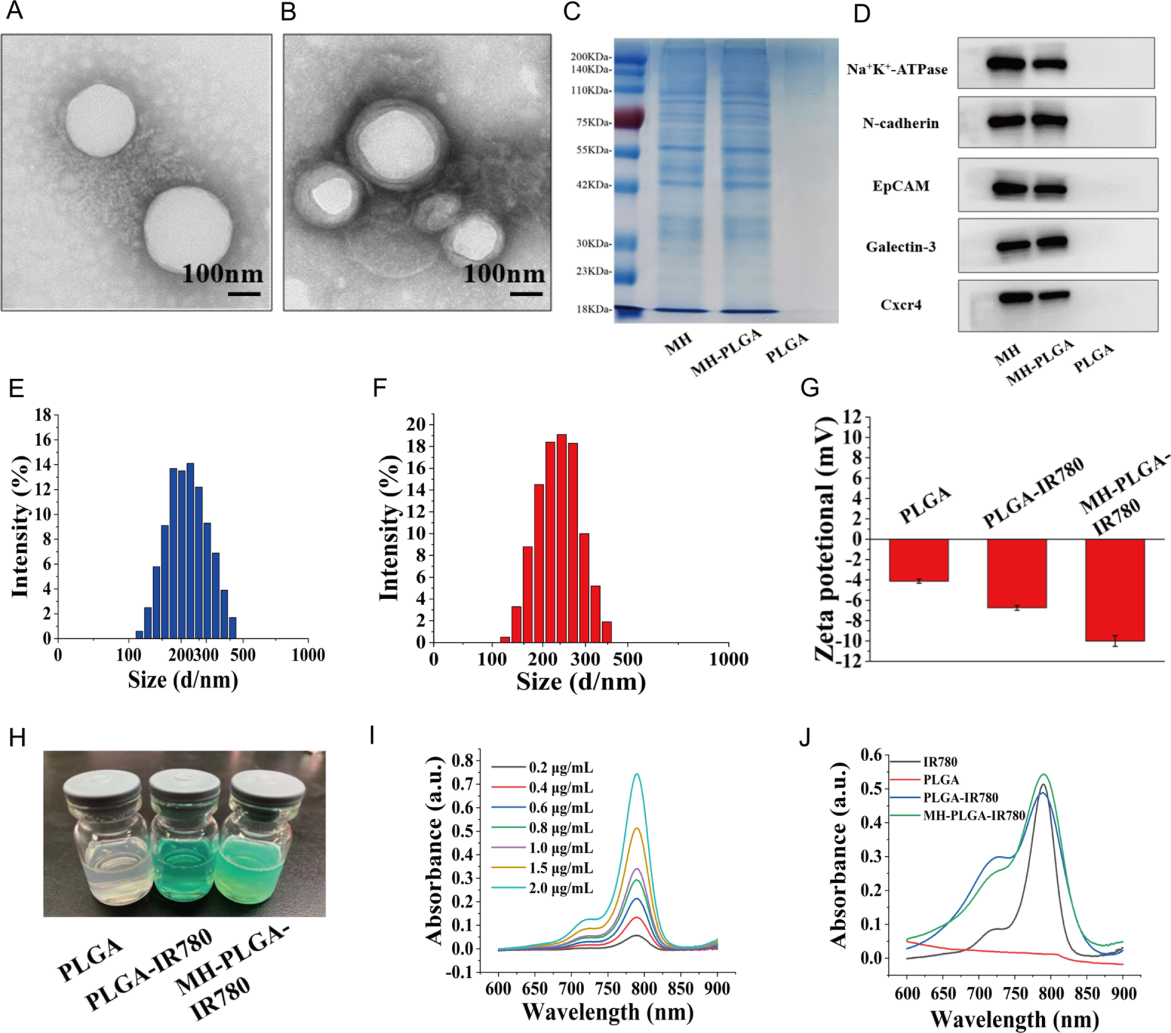
Characterizations of the MH-PLGA-IR780 NPs. **A**, **B** TEM images of PLGA NPs and MH-PLGA NPs. (scale bar: 100 nm). **C** SDS-PAGE protein analysis results. **D** Western blot analysis of membrane-specific protein markers. **E**, **F** Size distributions of the PLGA-IR780 NPs and MH-PLGA-IR780 NPs were measured by DLS. **G**, **H** Zeta potential (n = 3) and digital images of different NPs (PLGA NPs, PLGA-IR780 NPs, and MH-PLGA-IR780 NPs). **I** Absorbance spectra of IR780 in a concentration-dependent manner as recorded by UV–vis spectroscopy. **J** UV–vis spectra of single IR780, PLGA NPs, PLGA-IR780 NPs, and MH-PLGA-IR780 NPs

The morphology of the obtained NPs was observed by TEM (Fig. 1A, B). We found that all of the NPs were uniform in size and monodispersed, and that there was a lipid shell on the outside of the NPs wrapped by the cell membrane. Then, we analyzed the overall protein components in the PLGA NPs, MH, and MH-PLGA NPs by sodium dodecyl sulfate–polyacrylamide gel electrophoresis (SDS-PAGE). As shown in Fig. 1C, compared with the lack of protein expression in NPs without cell membrane coating, the expression levels of proteins in the MH and MH-PLGA NPs groups were comparable, which further confirmed successful encapsulation in the HOS cell membrane. To verify the presence of specific homologous-binding adhesion molecules on MH-PLGA-IR780 NPs, we examined the expression of cell membrane markers and some cellular adhesion molecules (Na^+^/K^+^-ATPase, Ncadherin, Galectin-3, EpCAM, and Cxcr4) for source-cell-specific targeting via the homologous binding mechanism by western blot analysis. As shown in Fig. 1D, the aforementioned proteins in the MH-PLGA-IR780 NPs group had similar expression to the cell membrane vesicle group, while NPs without cell membrane coating did not possess a protein profile, which demonstrated the stability and effectiveness of cell membrane proteins on MH-PLGA-IR780 NPs by extrusion coating. The particle sizes and zeta potentials of these NPs were measured by DLS, and the average sizes of PLGA-IR780 NPs and MH-PLGA-IR780 NPs were distributed at approximately 218.2 nm and 236.8 nm, respectively (Fig. 1E–F), which is consistent with the size determined by TEM. DLS also showed that the average sizes of the PLGA NPs were narrowly distributed and centered at 202.1 nm (Additional file 1: Fig. S1A), suggesting that the resulting average sizes of these NPs would allow them to pass through the tumor endothelial space [26]. Next, to examine the stability of the synthesized NPs, they were placed in either DMEM (10% serum) or PBS at 4 °C for one week. Their mean size measured every day did not change significantly within the 7-day analysis period (Additional file 1: Fig. S1B–C), suggesting that the obtained NPs had good stability under physiological conditions. In addition, the zeta potentials of the PLGA NPs and PLGA-IR780 NPs were − 4.62 ± 0.79 mV and − 6.70 ± 0.50 mV, respectively, while that of the MH-PLGA-IR780 NPs decreased to − 10.09 ± 0.70 mV (Fig. 1G), which was possibly caused by the negative charge from the cell membrane and the minimization of the electrostatic repulsion of IR780. The negative zeta potential of the MH-PLGA-IR780 NPs is beneficial for accurate tumor targeting due to a decrease in rapid elimination by the reticuloendothelial system (RES) and prolongation of the time in the systemic circulation [27]. The color of PLGA NPs and NPs-coated IR780 changed from white to green (Fig. 1H). The UV–vis spectrum showed that the PLGA-IR780 NPs and MH-PLGA-IR780 NPs had a characteristic absorption peak at 798 nm from IR780, further indicating the successful loading of IR780, unlike the PLGA NPs, which did not show this characteristic absorption peak (Fig. 1J). The absorbance of IR780 changed in a concentration-dependent manner, as determined by UV–vis spectroscopy, and a calibration curve was constructed (Figs. 1I, Additional file 1: S1D). Finally, the EE and LC of IR780 were calculated to be 67.8% and 3.25 wt%, respectively.

### Biosafety assessment of the MH-PLGA-IR780 NPs

**Fig. 2:**
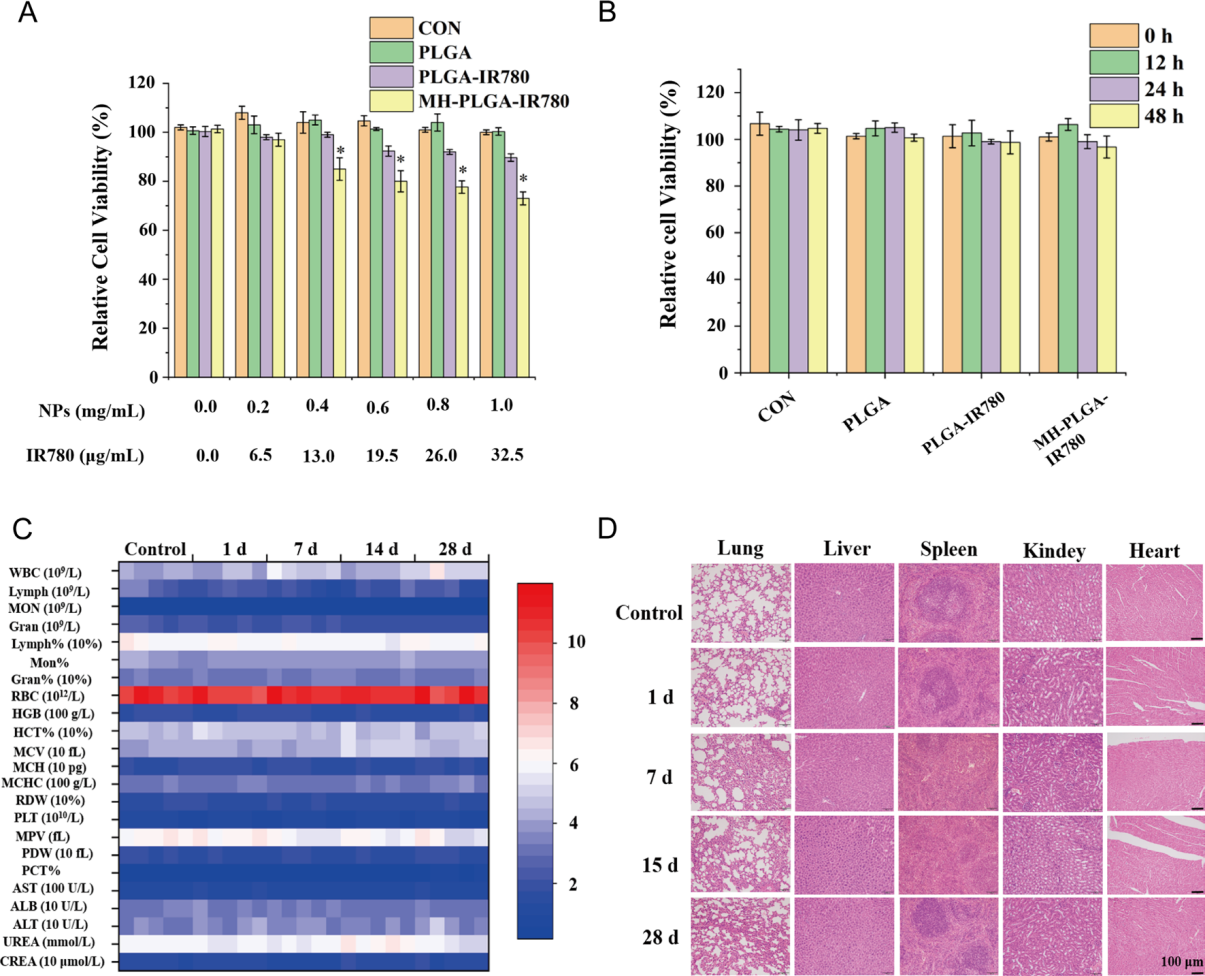
Biosafety of the MH-PLGA-IR780 NPs. **A** Relative viability (%) of HOS cells after coincubation with a wide range of NPs concentrations. **B** Relative viability (%) of HOS cells after coincubation with MH-PLGA-IR780 NPs (PLGA: 0.2 mg/mL) at prolonged time points. (The data are presented as the mean ± SD). **C**, **D** Blood indexes (routine blood and biochemistry) and H&E staining of the main organs (heart, liver, spleen, lung and kidney) of BALB/c nude mice were collected at 0, 1, 7, 14, and 28 days after post injection of MH-PLGA-IR780 NPs. (n = 5). The scale bars represent 100 µm

To verify the safety of the MH-PLGA-IR780 NPs in vitro, a CCK-8 assay was used to examine cell viability 12 h after coincubation of a wide range of concentrations of different NPs (PLGA: 0.0, 0.2, 0.4, 0.6, 0.8, 1.0 mg/mL) with HOS cells. As shown in Fig. 2A, the PLGA NPs had no toxicity in HOS cells, whereas IR780-encapsulating NPs exhibited weak cytotoxicity at a higher concentration (PLGA-IR780 NPs ≥ 0.6 mg/mL) because of the potential cytotoxicity of IR780, which was more obvious in the MH-PLGA-IR780 NP (NPs ≥ 0.4 mg/mL) treatment [28]. Then, a neutralized concentration of the NPs (PLGA: 0.2 mg/mL) was used to determine the cell viability at different treatment times (0, 12, 24, 48 h). The CCK-8 results demonstrated that the cell viability did not change with increasing coincubation time. Additionally, there are a large number of ligands and receptors on the surface of the cancer cell membranes that might cause biosafety risks, so MH-PLGA-IR780 NPs (PLGA: 5 mg/mL, 200 μL) were intravenously injected into BALB/c nude mice, and blood samples and major organs (heart, liver, spleen, lung and kidney) were harvested at 0, 1, 7, 14, and 28 days post injection. The routine blood examination and serum biochemical index results showed no significant differences between the samples, and no obvious histopathological changes were observed in the aforementioned vital organs after treatment with the MH-PLGA-IR780 NPs, indicating acceptable biocompatibility and biosafety in vivo (Fig. 2C, D).

### Homologous targeting improves intracellular uptake of the MH-PLGA-IR780 NPs

**Fig. 3:**
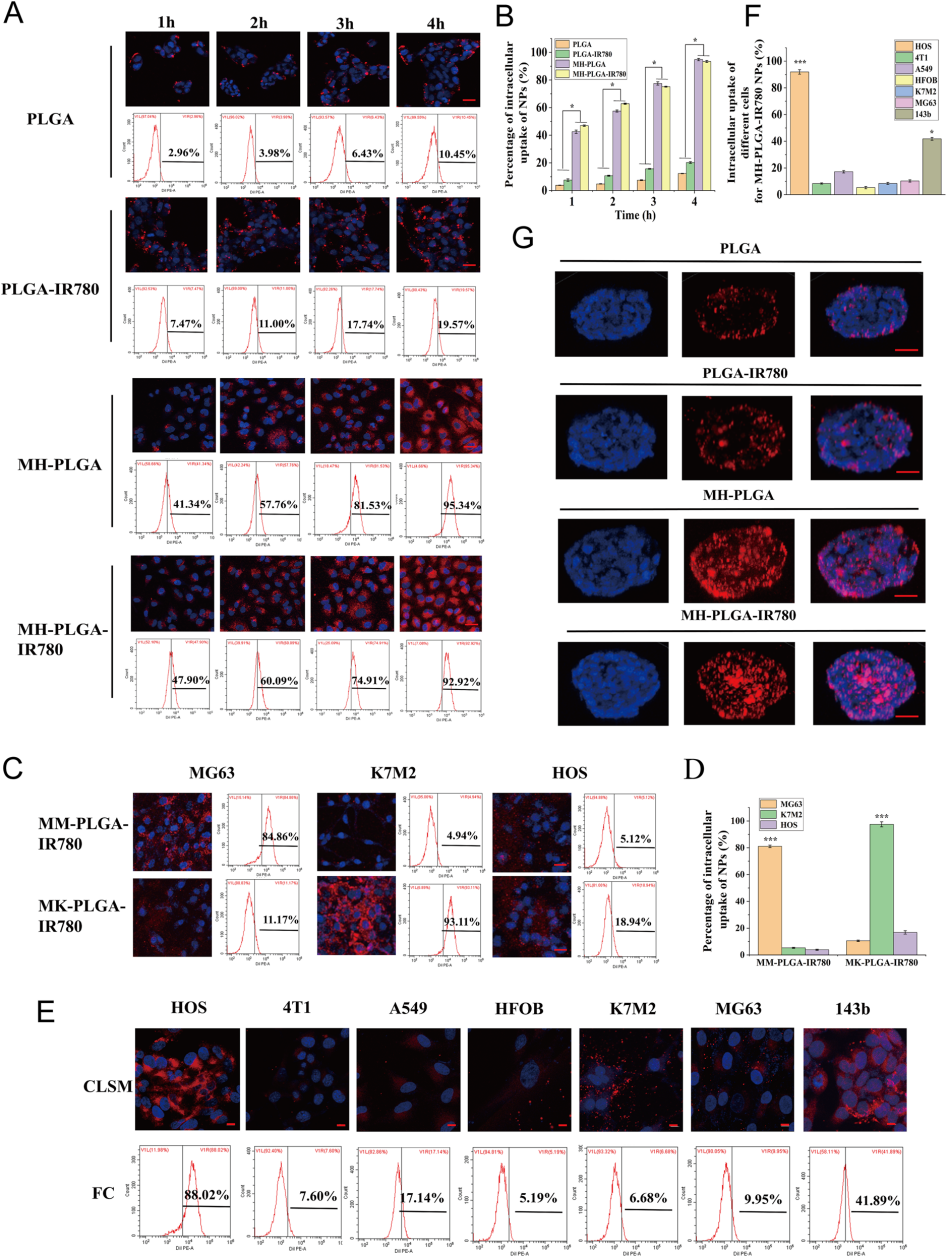
**A**, **B** Intracellular uptake of PLGA NPs, PLGA-IR780 NPs, MH-PLGA NPs and MH-PLGA-IR780 NPs (labelled with DiI) in the HOS cell line as measured by CLSM and FC analysis at prolonged coincubation times. The scale bars are 50 µm. Statistical analyses of the intracellular uptake rate of the four NPs. (The data are presented as the mean ± SD value, n = 3, *p < 0.05). **C**, **D** Homologous targeting capacity of PLGA-IR780 NPs wrapped by other OS (MG63, K7M2) cell membranes. The scale bars represent 50 µm. Statistical analyses of the intracellular uptake rate of the MK-PLGA-IR780 and MM-PLGA-IR780 NPs (labelled with DiI) to MG63, K7M2 and HOS cell lines. (The data are presented as the mean ± SD values, n = 3, *p < 0.05). **E**, **F** The homologous targeting capacity of MH-PLGA-IR780 NPs (labelled with DiI) was verified by coincubation with HOS, 4T1, A549, HFOB, K7M2, MG63 and 143b cell lines for 4 h. The scale bars represent 10 µm. Statistical analyses of the intracellular uptake rate of MH-PLGA-IR780 NPs to HOS, 4T1, A549, HFOB, K7M2, MG63 and 143b cell lines. **G** The penetration capability of PLGA NPs, PLGA-IR780 NPs, MH-PLGA NPs and MH-PLGA-IR780 NPs in HOS 3D tumor spheroids was observed by CLSM. The scale bars represent 50 µm

The essence of homologous targeting is ligand-receptor interactions based on the overexpressed surface antigens of cancer cell membranes. After binding to the target, the constructed NPs will be internalized into the cell through ligand-receptor-mediated endocytosis [15, 29]. In summary, homologous targeting narrows the time of intracellular endocytosis and contributes to effective phagocytosis. Therefore, to determine whether the MH-PLGA-IR780 NPs possessed high affinity for the HOS cell line, the cellular uptake efficiency of the MH-PLGA-IR780 NPs (labelled with DiI) was explored by CLSM and FC. As shown in Fig. 3A, B, compared with no obvious change in red FL intensity in PLGA NPs and PLGA-IR780 NPs, the red FL intensity in the cells coincubated with MH-PLGA NPs and MH-PLGA-IR780 NPs increased significantly with prolonged incubation time, with an uptake rate of greater than 90% after 4 h of coincubation, as determined by quantitative analysis by FC, indicating that encapsulating the NPs with the HOS cell membrane could solve the problem of the poor affinity of the traditional NPs for the HOS cell line.

Due to tumor heterogeneity, the expression levels of surface antigens in the cell membranes responsible for multicellular aggregation formation in tumors are relatively diversified. The cancer cell membrane should only have homologous targeting ability to the same cell line to confirm that homologous targeting indeed occurs in an OS cell lines. On the one hand, the OS cell lines MG63 and K7M2 were used to prepare the corresponding OS cell membranes coated with PLGA-IR780 NPs (MM-PLGA-IR780 NPs, MK-PLGA-IR780 NPs) to examine homologous targeting ability. Three OS cell lines (MG63, K7M2, and HOS) were individually coincubated with MM-PLGA-IR780 NPs and MK-PLGA-IR780 NPs (labelled with DiI), imaged using CLSM, and quantitatively analyzed by FC. As shown in Fig. 3C, D, CLSM showed that MM-PLGA-IR780 NPs only exhibited a strong red FL intensity in the MG63 cell line, while HOS and K7M2 cell lines showed weak FL intensities after coincubation with MM-PLGA-IR780 NPs. FC analysis further demonstrated that the intracellular uptake rate of the MG63 cell line reached 84.86%, while that of the K7M2 and MG63 cell lines was 4.94% and 5.12% after coincubation with MM-PLGA-IR780 NPs. In addition, only K7M2 presented an obvious red FL intensity after coincubation with MK-PLGA-IR780 NPs, the intracellular uptake rate of which reached 93.11%. However, the other two OS (MG63, HOS) cell lines showed weak red FL intensities, and the intracellular uptake rates were 11.17% and 18.94%, respectively. These results revealed the good binding ability of MM-PLGA-IR780 NPs and MK-PLGA-IR780 NPs to the original source of OS cell lines.

On the other hand, MH-PLGA-IR780 NPs should only exhibit a homologous targeting ability to the HOS cell line owing to their functionalization by adhesion proteins from the HOS cell membrane. To verify this hypothesis, PLGA-IR780 NPs with HOS cell membrane coating were coincubated with different tumor cell lines (human non-small-cell lung cancer A549 and murine breast cancer 4T1cells), other OS cell lines (MG63, 143b, K7M2) and human osteoblasts (HFOB 1.19). As shown in Fig. 3E, CLSM images showed that the red FL intensity (MH-PLGA-IR780 NPs) rose and gathered dramatically after 4 h of coincubation with the HOS cell line. Quantitative analysis showed an intracellular uptake rate by FC of 88.02%, while unsatisfactory red FL intensities were observed in the 4T1 and A549 cell lines, with intracellular uptake rates of 7.60% and 17.14%, respectively. Moreover, only a suboptimal increase (41.89%) was found in the red FL intensity in the 143b cell line, potentially because the 143b cell line is an HOS cell line with k-ras oncogenic transformation [30]. Poor intracellular uptake was observed in MG63 (9.95%), K7M2 (6.68%), and HFOB 1.19 (5.19%) cells (Fig. 3E, F), indicating that the PLGA-IR780 NPs wrapped by HOS cell membranes only have the potential ability to achieve tumor targeting in the HOS cell line. Collectively, these results confirmed that the homologous targeting ability of OS cell membrane-coated NPs depends on the source of the OS cell lines.

Moreover, hypertension at tumor sites caused by tumor vascular heterogeneity and an increase in the interstitial pressure further limit the penetration depth and width of NPs in the tumor region [31]. It has been reported that NPs with homologous targeting capacity can achieve surface-to-core penetration of tumor cells [16, 32]. Thus, the following experiments were conducted to evaluate whether the cancer cell membrane coating was desirable to improve the deep penetration ability of the PLGA-IR780 NPs, which was implemented using 3D tumor sphere models to simulate the complex conditions of tumor sites. We cultured HOS 3D tumor spheroids in vitro to examine the penetration capability of the different NPs. As shown in Fig. 3G, CLSM observations demonstrated that PLGA NPs and PLGA-IR780 NPs (labelled with DiI) failed to exhibit deep penetration capability, while the NPs with a cell membrane coating penetrated into the center of the HOS 3D tumor spheres for uniform distribution throughout the tumor cells, suggesting that the superior penetration depth and width of NPs in HOS 3D tumor spheroids can be achieved by homologous targeting. Furthermore, the immune escape capability of different NPs was evaluated in RAW 264.7 murine macrophage-like cells. RAW 264.7 cells were incubated with various NPs for 4 h and then imaged with CLSM. The red FL intensity in the PLGA NPs group was brighter than that in the PLGA-IR780 NPs group, while the MH-PLGA-IR780 NPs could avoid phagocytosis by RAW 264.7 cells and showed the least intense red FL intensity (Additional file 1: Fig. S2), indicating that coating NPs with the cancer cell membrane can endow them with the antiphagocytic capability of RAW 264.7 cells, possibly due to the reduction in immune clearance. In conclusion, the MH-PLGA-IR780 NPs achieved tumor targeting of HOS cells via homologous targeting by virtue of ligand receptors on the cell membrane.

### In vivo biodistribution by FL imaging

**Fig. 4:**
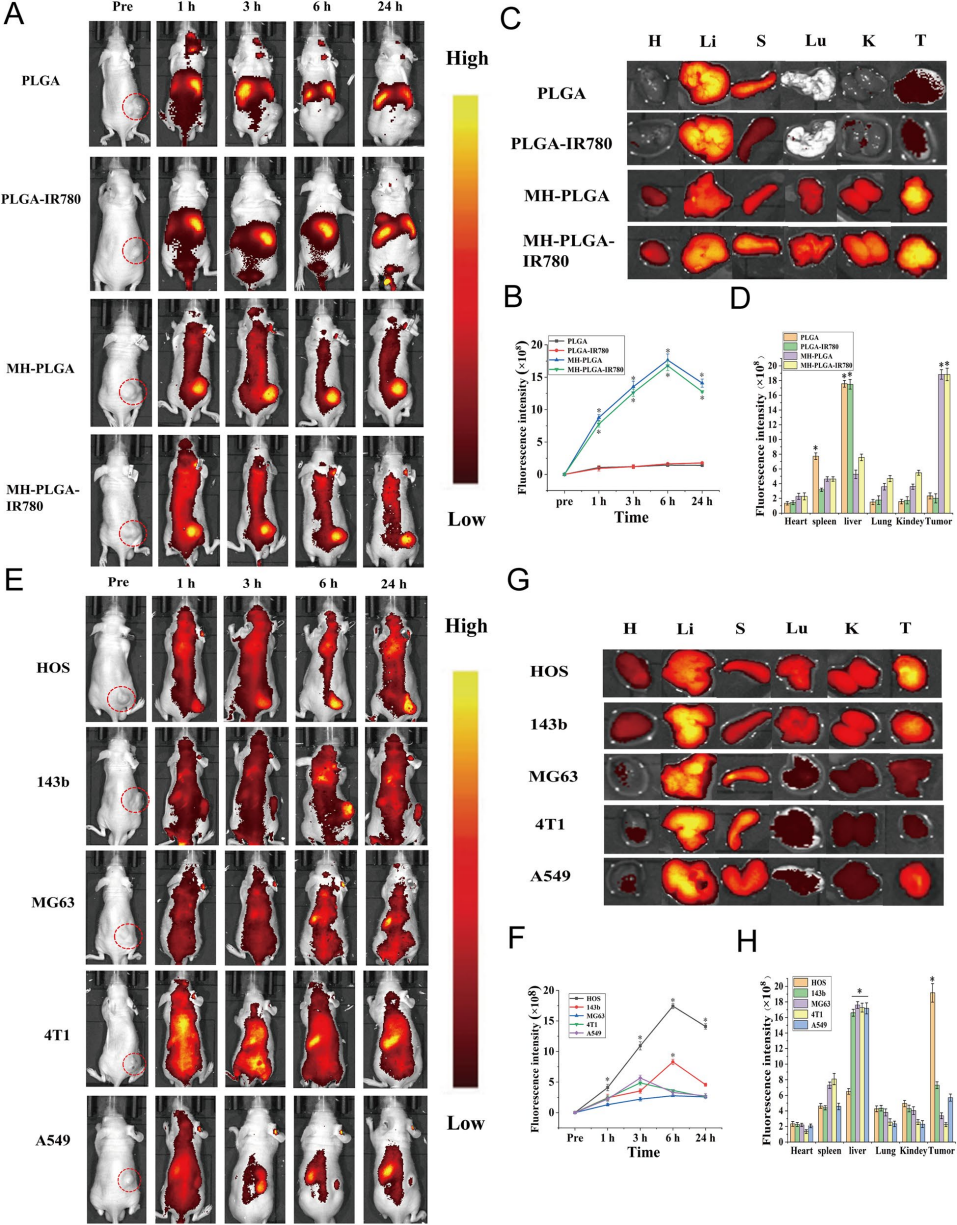
FL imaging of the different NPs in vivo. **A**, **B** FL images of HOS tumor-bearing mice and quantitative FL signal intensities within tumor regions before and after intravenous injection of different NPs at prolonged time points. **C** Ex vivo FL imaging of the tumor and major organs harvested from mice at 6 h post injection. **D** Semiquantitative biodistribution of tumors and major organs ex vivo was measured by the average FL intensities. (The data are presented as the mean ± SD values, n = 3, *p < 0.05). Biodistribution of MH-PLGA-IR780 NPs after intravenous injection in different tumor-bearing mice. **E**, **F** FL images and quantitative FL signal intensities of tumor regions at corresponding time points in vivo. (The data are presented as the mean ± SD values, n = 3, *p < 0.05). **G**, **H** Ex vivo FL images and semiquantitative analysis of major organs and tumors harvested from mice 6 h post-injection of MH-PLGA-IR780 NPs. (The data are presented as the mean ± SD values, n = 3, *p < 0.05)

IR780 has been used as a fluorescent probe to impart NPs with NIR FL (λ excitation/λ emission = 745 nm/820 nm) imaging capabilities to evaluate their biodistribution in vivo, which can further achieve real-time monitoring of their dynamic distribution to ensure satisfactory delivery for therapeutic performance [3]. Hence, different NPs were intravenously injected into HOS tumor-bearing mice to detect the biodistribution of the NPs in vivo after prolonged periods of time and to verify the optimal time for NPs to accumulate in tumors for further PDT. As shown in Fig. 4A, B, NPs without cell membrane coating mainly accumulated in the liver and spleen 24 h post injection, the two primary organs of the phagocyte-enriched reticuloendothelial system (RES) [33]. Compared with PLGA NPs and PLGA-IR780 NPs, the strongest FL signals began to aggregate within the tumor regions due to the accumulated NPs (MH-PLGA NPs and MH-PLGA-IR780 NPs) in a time-dependent manner and peaked at 6 h post injection. Then, the FL intensity at tumor sites gradually weakened. Moreover, the vital organs and tumors of mice were harvested 6 h post-injection for ex vivo FL imaging to further confirm the biodistribution of different NPs, and it was found that the FL intensities of NPs wrapped by cell membrane in tumors were higher than those of the heart, liver, spleen, lung and kidney (Fig. 4C, D), which could be attributed to the “homing affinity” of cancer cell membrane to tumor sites in vivo. However, the FL intensities of NPs without cell membrane coating mainly accumulated in the liver and spleen, exhibiting the same phenomenon as the FL signals in vivo and suggesting the superior targeting properties of NPs with cell membrane coating in vivo.

It has also been reported that homologous tumor-targeting ability in vivo reaches a peak at 24 h in human breast cancer MCF-7 cells [32, 34]. In general, the differential expression of cancer cell membrane proteins possesses separate intercellular homologous binding capabilities, which could be utilized for NPs surface functionalization to offer the advantage of complete replication of surface antigenic diversity. However, the specific homologous binding mechanism in OS was unclear and is the main focus of our next study.

### In vivo biodistribution by PA imaging

**Fig. 5:**
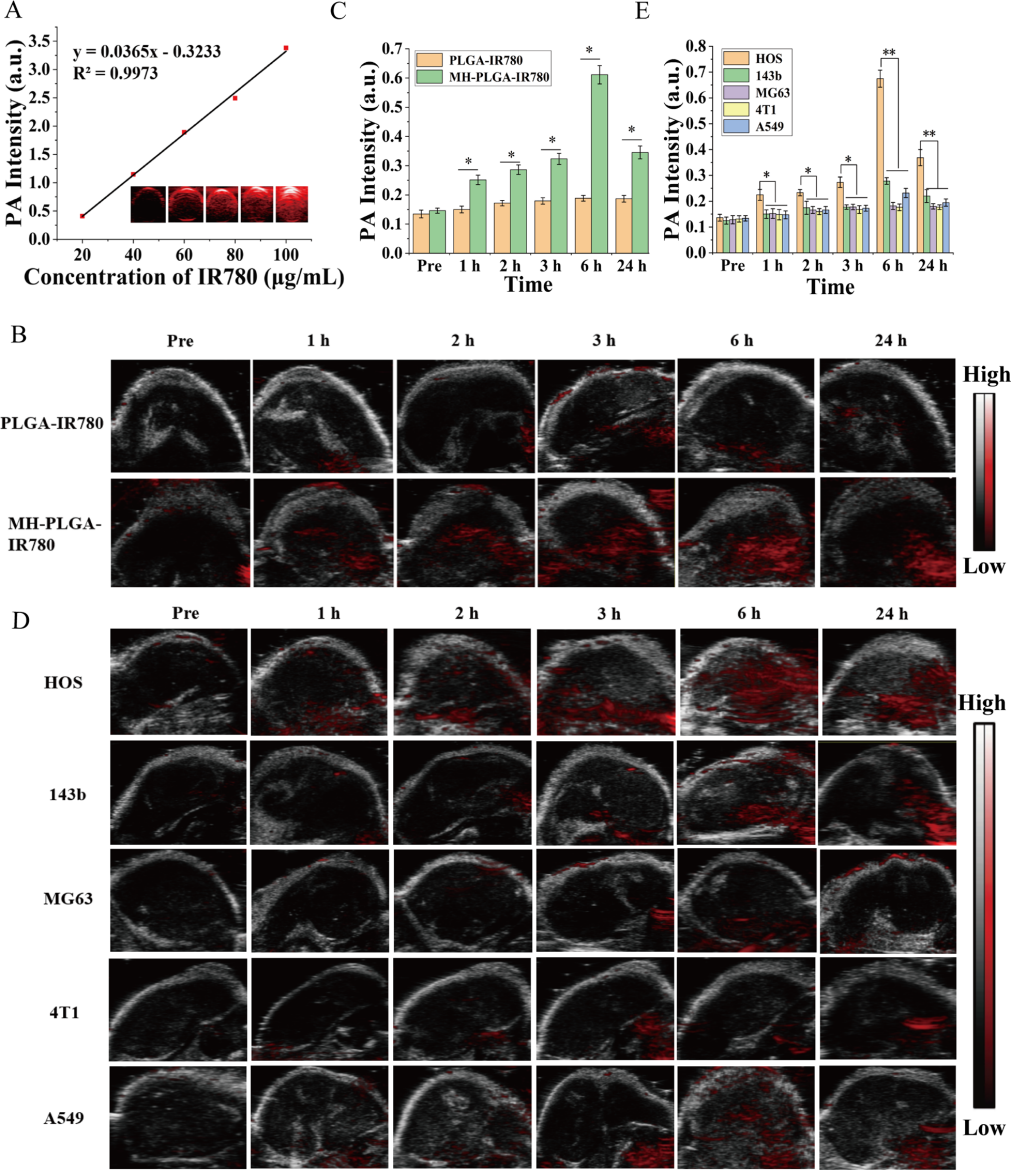
**A** PA images and the corresponding signal intensities of MH-PLGA-IR780 NPs at various concentrations. **B**, **C** PA images and quantitative PA signal intensities of tumor regions before and after intravenous administration of PLGA-IR780 NPs and MH-PLGA-IR780 NPs at corresponding time points. (The data are presented as the mean ± SD values, n = 3, *p < 0.05). **D**, **E** PA image and quantitative PA signal intensities of different tumor-bearing mice before and after the injection of PLGA-IR780 NPs and MH-PLGA-IR780 NPs. (The data are presented as the mean ± SD values, n = 3, *p < 0.05, **p < 0.01)

In biological tissues, PA imaging has superior contrast, resolution and penetration, enabling the integration of diagnosis and treatment by determining the precise location of the drugs in the targeted region of the tumor [35]. Therefore, PA imaging was recorded to further clarify the distribution of the MH-PLGA-IR780 NPs in vivo. According to related research, the optimal excitation wavelength of NPs coating IR780 for PA imaging was approximately 800 nm. Then, the PA signal of NPs was measured clearly in a dose-dependent manner with an excellent linear relationship (Fig. 5A), increasing from 0.410 to 3.382 with increasing NPs concentration (5, 10, 20, 40, and 80 μg/mL). For PA imaging in vivo, an obvious PA signal intensity began to focus on the tumor region due to the accumulated MH-PLGA-IR780 NPs at 1 h post injection and peaked at 6 h post injection, but only a weak PA signal in tumor tissue was observed in the mice treated with PLGA-IR780 NPs (Fig. 5B, C), consistent with the FL signals in vivo.

To further demonstrate the homologous targeting ability of MH-PLGA-IR780 NPs to xenograft tumors in vivo, MH-PLGA-IR780 NPs were intravenously injected separately into HOS, 143b, MG63, 4T1, and A549 tumor-bearing nude mice, and FL and PA images were then acquired and quantitatively analyzed. As shown in Figs. 4E, F and 5D, E, obvious FL and PA signals were observed in the tumor regions of HOS xenograft tumors even at 1 h post injection and reached a maximum at 6 h post injection. However, MG63, 4T1 and A549 tumor-bearing mice exhibited weak FL and PA intensities in tumor regions at each post injection time point. Owing to the k-ras oncogenic transformation, the FL and PA intensities in the tumor regions of 143b xenograft tumors were higher than those of MG63, 4T1, and A549 xenograft tumors, which also peaked at 6 h post injection. To further quantitatively analyze the biodistribution of MH-PLGA-IR780 NPs in different xenograft tumors, tumors and major organs of the sacrificed mice were harvested for ex vivo FL imaging 6 h post injection. The semiquantitative results showed that MH-PLGA-IR780 NPs mainly accumulated at the tumor sites of HOS xenograft tumors at levels approximately 2.8-, 5.1-, 5.7-, and 4.1-fold higher than those in the 143b, MG63, 4T1, and A549 xenograft tumors, respectively. The amount of MH-PLGA-IR780 NPs of the aforementioned xenograft tumors mainly accumulated in the liver, which was higher than those of HOS xenograft tumors, increasing by 2.6-, 2.8-, 2.8-, and 2.8-fold, respectively (Fig. 4G, H). In conclusion, the results of both FL and PA imaging verified that the MH-PLGA-IR780 NPs might effectively accumulate in the tumor region in vivo by homologous targeting, laying a foundation for further tumor diagnostic imaging and targeted PDT. Although the current work only used NPs with a HOS cell membrane coating to verify the homologous targeting ability in vivo, it is envisioned that the homologous targeting ability of other OS cell membrane coatings could be effective.

### Homologous targeting enhanced IR780-mediated PDT performance by mitochondrial dysfunction

**Fig. 6:**
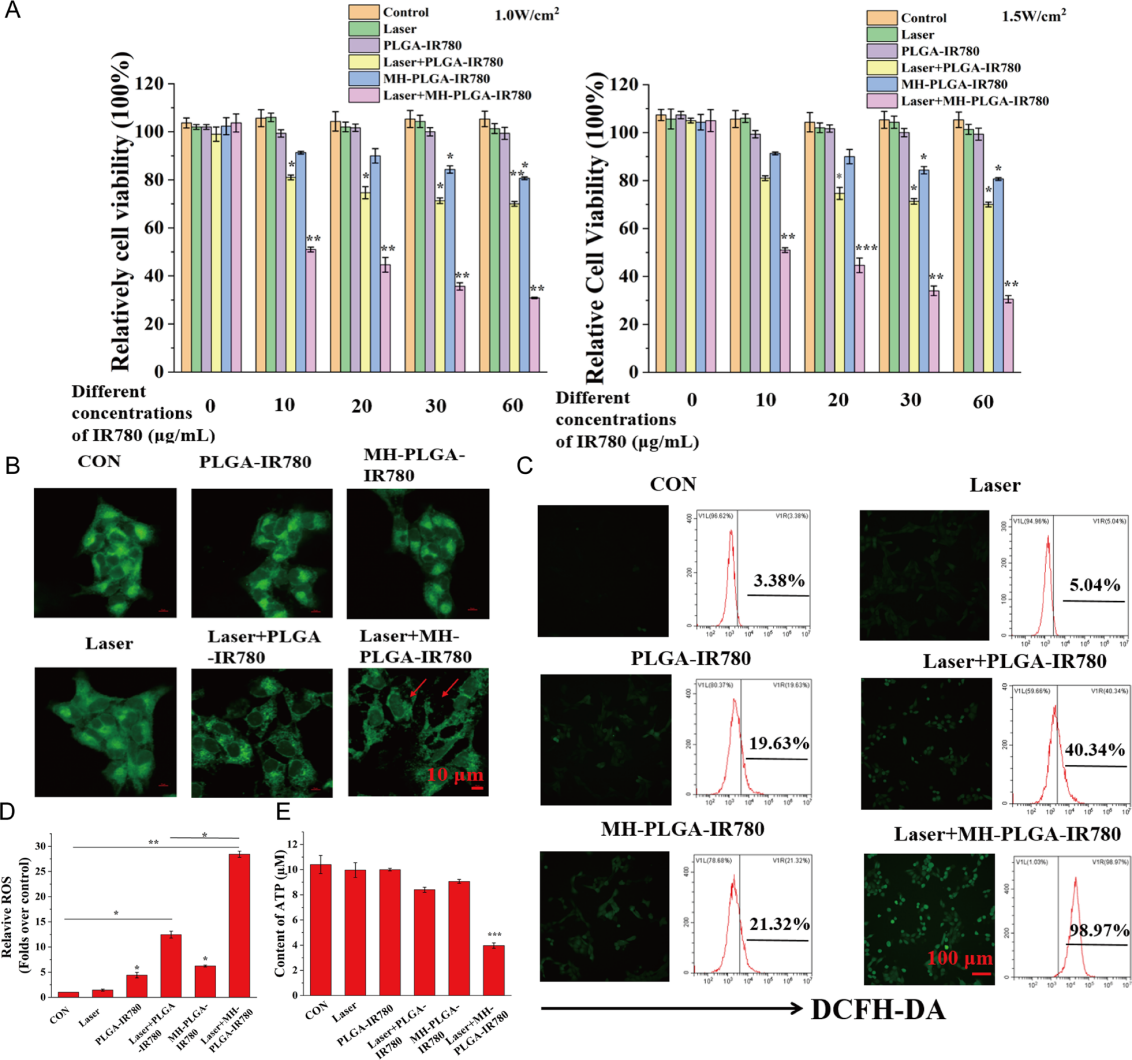
PDT performance with the assistance of homologous targeting. **A** Relative cell viability (%) of HOS by various treatments. **B** Aggravated mitochondrial fragmentation of HOS cells after targeted PDT. The scale bars represent 10 µm. **C**, **D** CLSM imaging and FC analysis of the production of ROS levels in HOS cells (stained with DCFH-DA) after different treatments. The scale bars represent 100 µm. (The data are presented as the mean ± SD values; n = 3, *p < 0.05, **p < 0.01.) **E** The ATP content in HOS cells after different treatments (the data are presented as the mean ± SD values; n = 3, ***p < 0.001)

After tumor targeting was achieved by homologous targeting, a CCK-8 assay was used to estimate the antitumor efficacy of PDT in vitro. As shown in Fig. 6A, compared to no obvious change in the control, laser alone, and single NPs without NIR irradiation groups, the MH-PLGA-IR780 NPs exposed to laser (targeted PDT group) decreased the viability of HOS cells in an IR780 dose-dependent manner at power densities of 1 W/cm^2^ and 1.5 W/cm^2^, while laser + PLGA-IR780 NPs (PDT without targeting group) showed weak photoinduced cytotoxicity in HOS cells. Considering that the use of single MH-PLGA-IR780 NPs alone at a concentration greater than 10 μg/mL had weak cytotoxic effects on HOS cells after 12 h of coincubation, MH-PLGA-IR780 NPs (5 μg/mL) were selected to examine the efficacy and specific mechanism of MH-PLGA-IR780 NPs-mediated PDT in the following experiments.

First, the morphology of the mitochondria was observed by CLSM. As shown in Fig. 6B, compared with the physiological morphology of mitochondria in the other five groups, mitochondrial fragmentation was found in HOS cells upon targeted PDT, which could further lead to mitochondrial dysfunction. In addition, PDT performance was detected by DCFH-DA, a probe that detects intracellular ROS generation by showing green FL. As shown in Fig. 6C, D, compared with the lack of a significant alteration in the control and laser alone groups, PDT without targeting showed green FL intensity, while targeted PDT produced strong green FL intensity in HOS cells under 808 nm NIR irradiation (1.5 W/cm^2^), which was accompanied by typical morphological features of apoptotic cells. Moreover, we quantitatively analyzed the percentage of intracellular ROS by FC, and compared with the parallel groups, the amount of ROS produced by PDT without the targeting group was 40.34%, while this value after treatment with the targeted PDT group was significantly increased to 98.97%. Owing to the potential cytotoxicity of IR780 even without laser irradiation at high concentrations [28, 36], the two single NPs groups exhibited slight ROS accumulation (19.63% and 21.32%). ATP, one of the most important molecules in energy supply, is produced mainly via mitochondrial metabolism [37] and plays a key role in tumor proliferation and DNA replication. It has been reported that ATP depletion increases the sensitivity of tumor cells PDT [38]. To demonstrate the changes in ATP contents after different treatments, a calibration curve of ATP was constructed (Additional file 1: Fig. S3A). As shown in Fig. 6E, compared with the other five treatments, only PDT with homologous targeting led to a significant decrease in intracellular ATP content. In conclusion, these results suggested that homologous targeting-based NPs could improve the performance of PDT by increasing intracellular ROS production and inhibiting ATP synthesis due to mitochondrial dysfunction.

### MH-PLGA-IR780 NPs-mediated PDT synergistically induced apoptosis and ferroptosis

Cell death is a fundamental biological process that generally including necrosis, apoptosis, autophagy and ferroptosis. However, the specific death mechanism of this homologous targeting NPs-guided PDT is unclear. Synergistic induction of multiple death modes in combination with tumor therapy is an effective strategy to improve PDT performance [19]. Therefore, a CCK-8 assay was performed to investigate the main cell death modes. Before NIR irradiation, HOS cells were pretreated with multiple inhibitors of cell death pathways, including an apoptosis inhibitor (z-VAD-FMK), a necrosis inhibitor (Nec-1), an autophagy inhibitor (Baf-A1), a ferroptosis inhibitor (Fer-1), and a general ROS scavenger (NAC), for 24 h. As shown in Additional file 1: Fig. S3B, the CCK-8 assay showed that compared with the significant inhibitory effect of PDT on cell viability, cell viability increased by different amounts after pretreatment with each inhibitor (except Baf-1) followed by PDT. However, NAC, Fer-1, and z-VAD-FMK significantly protected cells from photodynamic cytotoxicity, while Baf-1 promoted the inhibitory effects of PDT on cell viability, suggesting that in addition to apoptosis, ferroptosis may be another key cell death mode mediated by MH-PLGA-IR780 NPs-guided PDT and that autophagy plays a protective role in PDT.

**Fig. 7:**
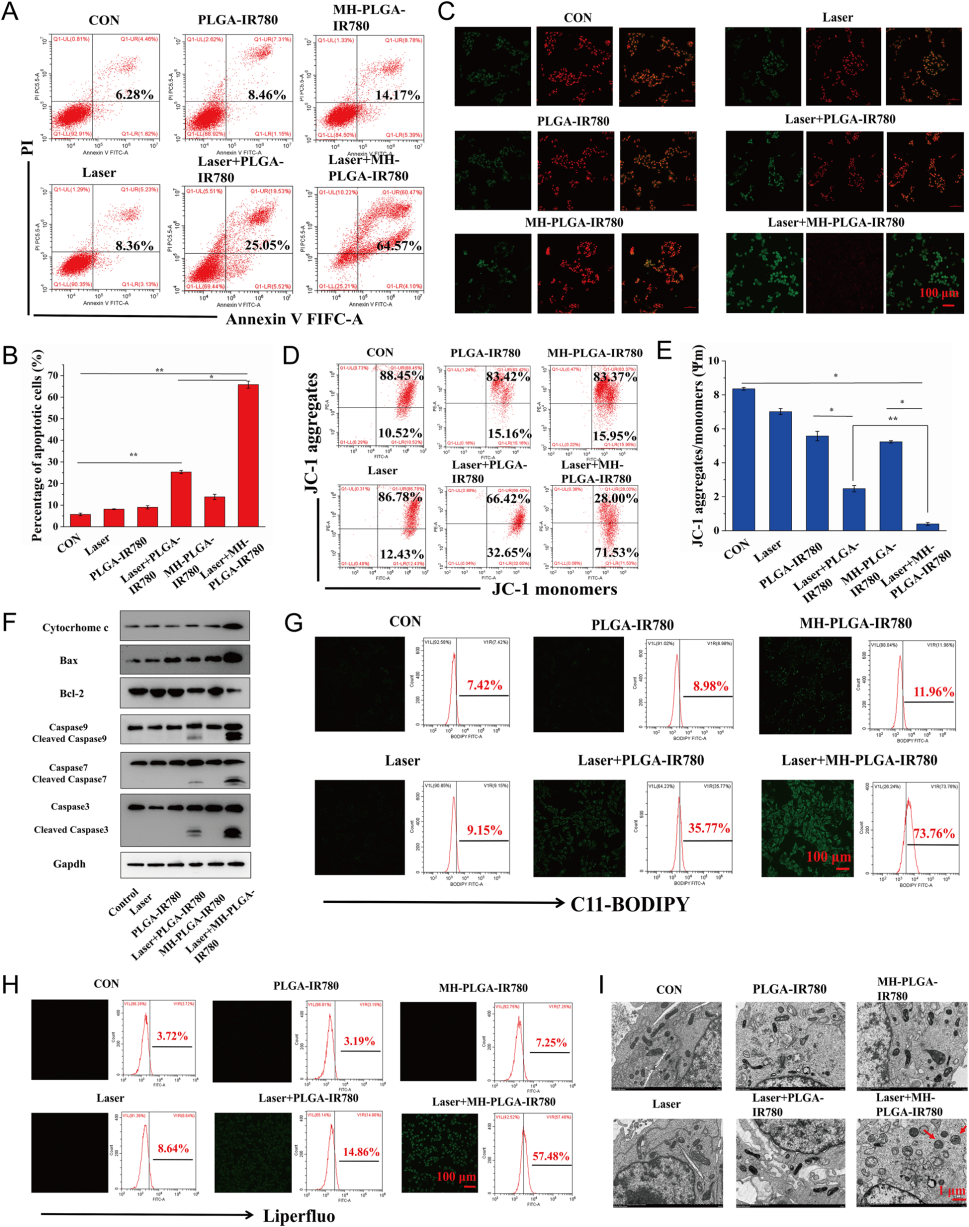
Evaluation of apoptosis and ferroptosis. **A, B** Induction of apoptosis in HOS cells (stained with annexin V-FITC/PI) after various treatments by FC analysis. (The data are presented as the mean ± SD values; n = 3, *p < 0.05, **p < 0.01.) **C**–**E** Changes in Δψm in HOS cells stained with JC-1 after various managements, as observed via CLSM and FC. (The data are presented as the mean ± SD values; n = 3, *p < 0.05, **p < 0.01). The scale bars represent 100 µm. **F** The expression levels of cell apoptosis-related proteins were measured by western blot analysis. **G**, **H** The excessive production of LPO and Lipid ROS in HOS cells after targeted PDT as measured by CLSM and FC. The scale bars represent 100 µm. **I** The morphology of mitochondria after various treatments as observed by TEM. The scale bars represent 1 µm

Therefore, we hypothesized that the constructed homologous targeting NP-mediated PDT is desirable to synergistically induce mitochondrial apoptosis and ferroptosis to kill HOS cells. To further verify this assumption and explore the underlying cell death mechanism, we first measured the HOS cell apoptosis rate by FC using annexin V-FITC/PI double staining. As shown in Fig. 7A, B, the control, NIR irradiation alone, and two NPs without irradiation groups did not exhibit obvious HOS cell apoptosis. The apoptosis rate of HOS cells induced by PDT without targeting was 25.05%, while targeted PDT increased the apoptosis rate to 64.57%. Then, considering the important role Δψm in mitochondrial apoptosis, we measured Δψm with the fluorescent probe JC-1. During mitochondrial apoptosis, mitochondrial membranes are disrupted, with depolarization of Δψm, and mitochondrial depolarization was verified by the decrease in the ratio of red to green FL intensity in the JC-1 assay. As shown in Fig. 7C, E, HOS cells were stained with JC-1 after different treatments. Compared with the lack of obvious change in the control, single NIR irradiation, and two NPs alone groups, a slight decrease in red FL intensity and a weak increase in green FL intensity were observed in the laser + PLGA-IR780 NPs group, and these changes were more prominent after treatment with laser + MH-PLGA-IR780 NPs. To further investigate the mechanisms underlying the proapoptotic effects of the engineered nanoplatform-mediated PDT, the expression levels of apoptosis-related proteins were assessed by western blot analysis. Laser + MH-PLGA-IR780 NPs treatment significantly increased the protein levels of cytochrome c, cleaved caspase-7, cleaved caspase-9, and the proapoptotic protein Bax, accompanied by an obvious downregulation of the antiapoptotic protein Bcl-2, which ultimately led to a marked increase in caspase-3 cleavage. However, the lack of a prominent change in the expression levels of these apoptosis-related proteins in the control, single NIR irradiation, NPs without irradiation, and PDT without targeting produced unstable pro-apoptotic effects (Fig. 7F, Additional file 1: Fig. S4).

Intriguingly, the homologous targeting NPs platform developed in our study have the innate advantage of inducing ferroptosis under NIR irradiation. On the one hand, the increase in intracellular ROS and the large number of polyunsaturated fatty acids (PUFAs) in the cell membranes were found to be the key drivers of ferroptosis [39], which are susceptible to oxidation to form LPOs. On the other hand, mitochondrial dysfunction-mediated ferroptosis has been reported to be an emerging strategy for cancer therapy [40]. To further examine the level of ferroptosis, LPOs and Lipid-ROS have been recognized as crucial biomarkers of ferroptosis to impair cell structure and integrity, and both were detected by liperfluo and C11-BODIPY, respectively [41]. As shown in Fig. 7G, H, CLSM images showed that the contents of both LPOs and Lipid ROS in HOS cells were significantly increased after laser + MH-PLGA-IR780 NPs treatment, as indicated by the significant increase in green FL intensity, compared with a lack of marked alteration in the control, laser alone, and two single NP without irradiation groups. In addition, weak green FL was observed after laser + PLGA-IR780 NPs treatment, which was consistent with the weak induction of apoptosis by this treatment. Moreover, quantitative analysis via FC illustrated the same phenomenon (Additional file 1: Fig. S5), indicating that this homologous targeting-based theranostic nanoplatform had superior advantages in inducing ferroptosis under NIR irradiation, and the inadequate ability of PLGA-IR780 NPs combined with NIR irradiation to induce ferroptosis may be due to the lack of mitochondrial dysfunction and PUFAs. Mitochondrial shrinkage is another important characteristic of the death phenotype in ferroptosis [42, 43]. The TEM results also demonstrated that the morphology of mitochondria treated with targeted PDT became rounded, decreased in size, exhibited reduced or absent mitochondrial cristae, and exhibited structural damage, while mitochondria in the other five groups showed a normal physiological morphology (Fig. 7I). Therefore, we confirmed that engineered nanoplatform-mediated PDT can significantly and synergistically induce HOS cell apoptosis and ferroptosis.

### Targeted PDT promoted ferroptosis by inactivating GPX4 and accumulating Fe^2+^ by ferritinophagy

**Fig. 8:**
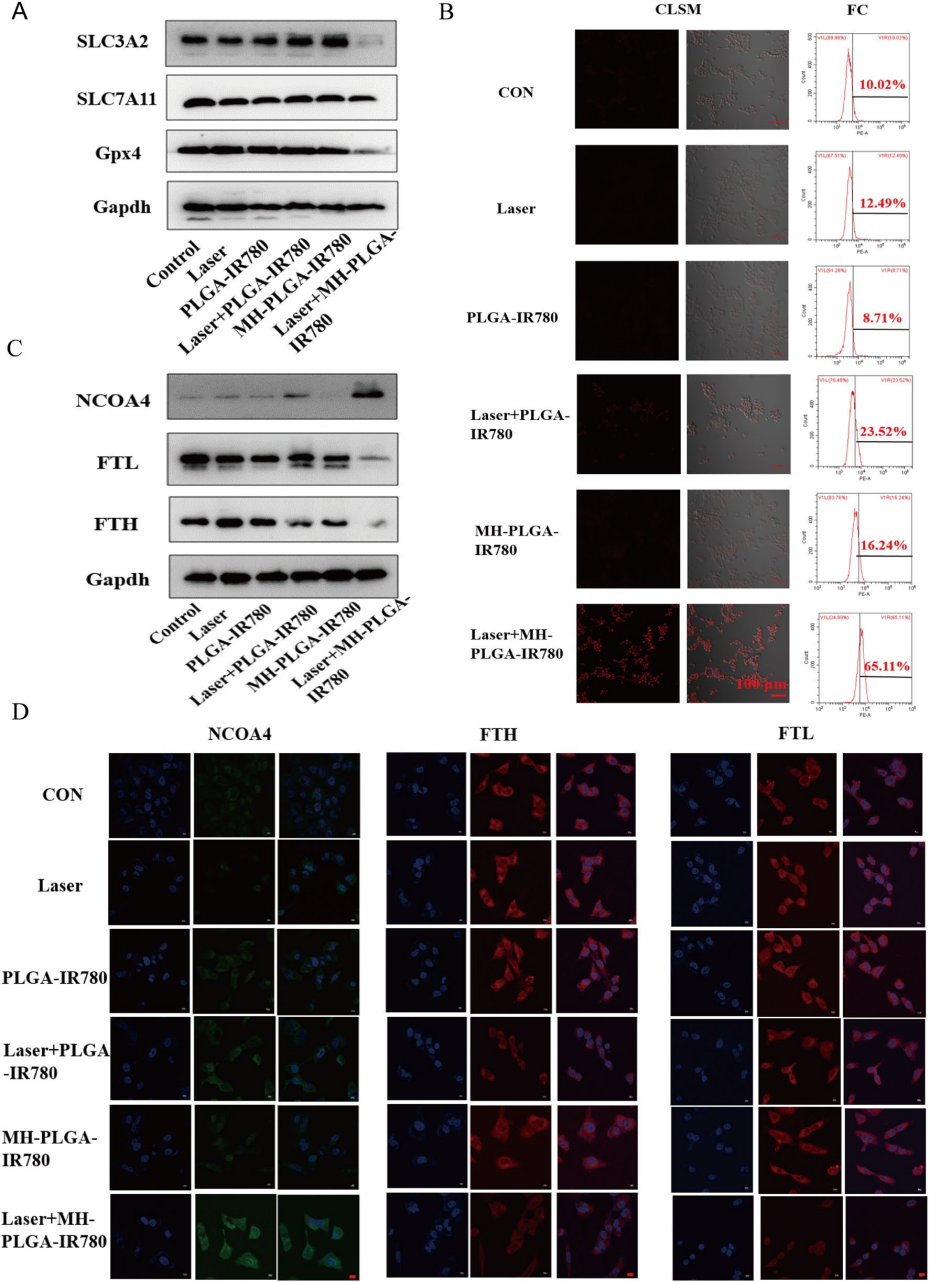
The specific mechanism of the induction of ferroptosis by targeted PDT. **A** The expression levels of ferroptosis-related proteins after different treatments detected by western blot. **B** Enhanced intracellular Fe^2+^ production of targeted PDT in HOS cells. CLSM images (labelled with FerroOrange) and FC analysis of Fe^2+^ generation after different treatments. The scale bars are 100 µm. **C** Western blot analysis of the expression levels of ferritinophagy-related proteins (NCOA4, FTH and FTL) after various treatments. **D** IF images of NCOA4, FTH and FTL in HOS cells after different treatments. The scale bars represent 20 µm

Three of the major factors that affect ferroptosis are tailored lipid metabolism, accumulation of redox-active iron (Fe^2+^), and inactivation of GPX4, a lipid repair enzyme that is responsible for preventing LPO cytotoxicity and maintaining membrane lipid bilayer homeostasis [20]. To further investigate the specific mechanism of ferroptosis induced by MH-PLGA-IR780 NPs-guided PDT, we first detected the protein expression of GPX4 by western blot analysis, as shown in Fig. 8A and Additional file 1: Fig. S6. GPX4 expression was significantly downregulated by laser + MH-PLGA-IR780 NPs treatment, while the other five treatments did not achieve identical effects. SLC7A11 and SLC3A2 are subunits of system X_C_^−^, a glutamate/cysteine antiporter that is responsible for maintaining the cellular antioxidant environment, and the low expression of both of these subunits would induce the inactivation of GPX4, preventing LPO production during ferroptosis [44]. Unsurprisingly, compared with the parallel treatment groups, PDT without targeting showed no influence on either SLC7A11 or SLC3A2 protein expression. However, SLC3A2 protein expression was significantly downregulated by laser + MH-PLGA-IR780 NPs treatment, and the protein expression of SLC7A11 was only slightly decreased in this group. Finally, downregulation of both SLC3A2 and SLC7A11 resulted in the deactivation of GPX4.

In addition, excessive accumulation of redox-active iron in cells induced by mitochondrial dysfunction promotes the production of excessive ·OH via the Fenton reaction (Fe^2+^  + H_2_O_2_ → Fe^3+^  + (OH)– + ·OH). ·OH, another ROS, is able to oxidize PUFAs, producing LPOs. Thus, we measured the intracellular Fe^2+^ generation capability of homologous targeting nanoplatform-associated PDT by FerroOrange (Japan, colleagues). As shown in Fig. 8B, CLSM analysis showed that compared with the parallel groups, the targeted PDT significantly increased the intracellular Fe^2+^ levels, as shown by the strong red FL intensity. The quantitative analysis of FC also confirmed that the percentage of intracellular Fe^2+^ resulting from the targeted PDT increased to 65.11%, which was consistent with the above findings (Additional file 1: Fig. S7A). It has been reported that nuclear receptor coactivator 4 (NCOA4)-mediated ferritinophagy increases the level of redox-active iron in the cytoplasm and induces ferroptosis by degrading ferritin [45, 46]. The proteins involved in iron storage are ferritin heavy chain (FTH) and ferritin light chain (FTL). To further analyze whether the specific molecular mechanism of the obtained increase in intracellular Fe^2+^ level was associated with NCOA4-mediated ferritinophagy, the protein expression levels of NCOA4, FTH, and FTL were determined by western blotting. As shown in Fig. 8C and Additional file 1: Fig. S7B–D, accompanied by the lack of obvious changes in the control, laser alone, and NPs without irradiation groups, the PDT without targeting group showed a slight increase in NCOA4 expression and a slight reduction in FTH expression, while the expression of NCOA4 was significantly elevated and the expression of FTH and FTL were markedly diminished in the laser + MH-PLGA-IR780 NPs group. In addition, the levels of FTH and FTL expression were further confirmed by immunofluorescence (IF) staining. Generally, consistent with the western blot results, the laser + MH-PLGA-IR780 NPs showed significant green FL intensity from NCOA4, which was brighter than that in the other five groups. Additionally, noticeably weak red FL intensities of FTH and FTL were observed in the laser + MH-PLGA-IR780 NPs group; these levels were markedly lower than those in the control, single NIR irradiation, and two NP without irradiation groups, while the PDT without targeting group showed limited changes in the FL intensities of NCOA4, FTH, and FTL (Fig. 8D). In conclusion, we demonstrated that this homologous targeting-associated theranostic nanoplatform not only can decrease the activity of GPX4 by inhibiting the system Xc^−^ transporter but also can promote the accumulation of Fe^2+^ by activating NCOA4-mediated ferritinophagy to degrade ferritin, synergistically inducing ferroptosis in HOS cells.

### Antitumor efficacy in vivo

**Fig. 9:**
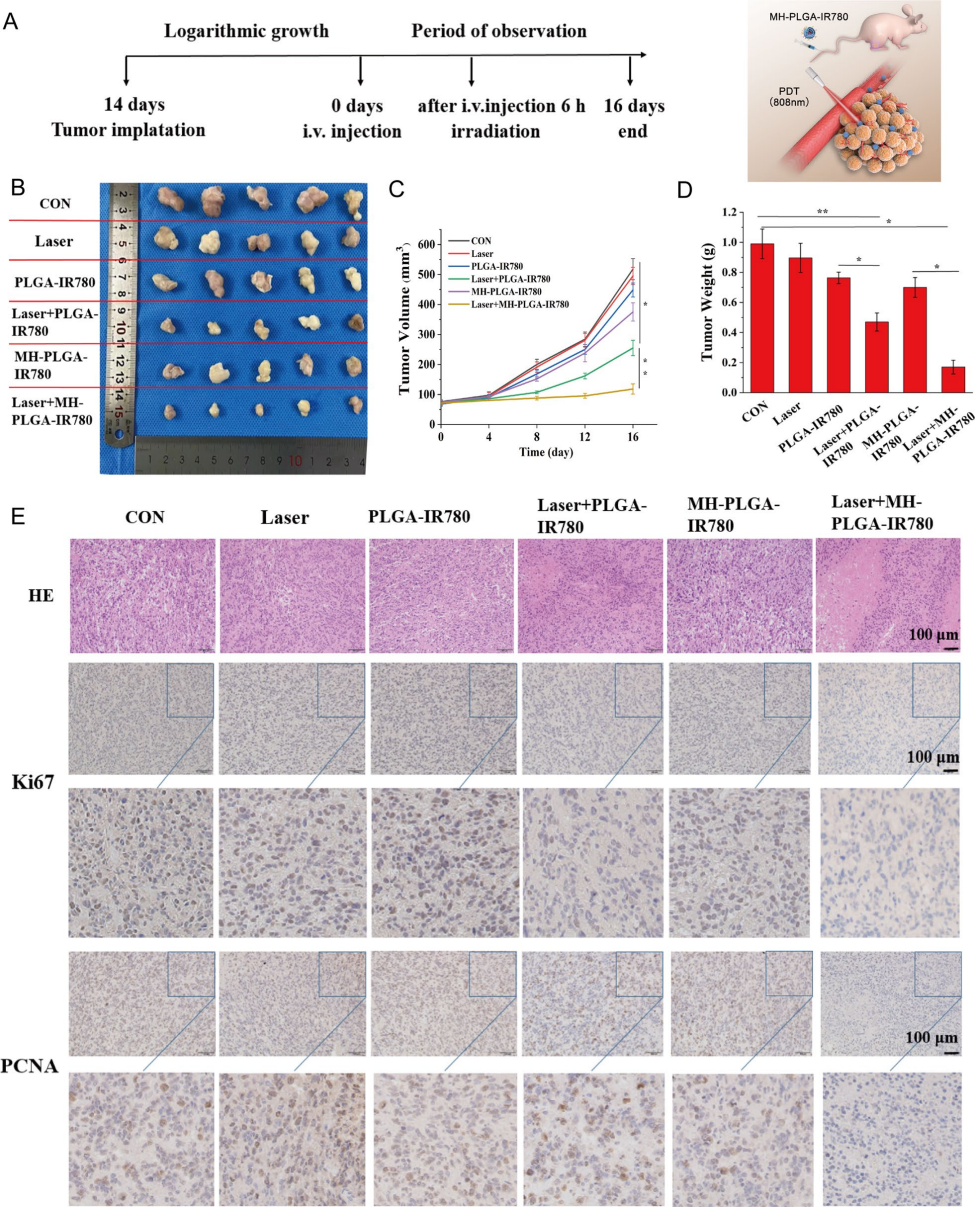
Antitumor capacity in vivo. **A** Schedule of the targeted PDT. **B** Photographs of tumors harvested from mice after various treatments (n = 5) and HOS tumor-bearing mice before sacrifice. **C** Tumor volume was measured during the therapeutic period. (The data are presented as the mean ± SD values; n = 5, *p < 0.05, **p < 0.01.) **D** Tumor weights were recorded at the end of therapy. (The data are presented as the mean ± SD values; n = 5, *p < 0.05, **p < 0.01). **E** H&E staining and images of PCNA and Ki67 immunohistochemical staining in tumor specimens after various treatments. The scale bars represent 100 µm

Based on the effective induction of cell apoptosis and ferroptosis by MH-PLGA-IR780 NPs-mediated PDT in vitro, we examined whether a similar inhibitory effect could occur in a xenograft model. HOS bearing mice were subcutaneously divided into six groups for different treatments: (1) control (PBS), (2) laser alone, (3) single PLGA-IR780 NPs, (4) laser + PLGA-IR780 NPs, (5) single MH-PLGA-IR780 NPs, and (6) laser + MH-PLGA-IR780 NPs. The tumor regions were irradiated with an NIR laser (808 nm, 2 W/cm^2^, 5 min) after 6 h post injection in the appropriate groups considering the PA/FL imaging results. After different therapies, the representative images of the mice before sacrifice and tumor visualization were captured, and tumor volumes were measured every 4 days over 16 days to monitor different treatment outcomes (Fig. 9A, B, Additional file 1: Fig. S8A). We found that tumor growth in the PBS and laser alone groups was increased nearly 6.82-fold and 6.13-fold compared with the original tumor volumes, the tumor volumes in the single two NPs groups presented 5.25-fold and 4.78-fold increases. Suboptimal tumor growth inhibition (indeed, a 3.07-fold increase in tumor volume) was observed in the laser + PLGA-IR780 NPs group due to the lack of targeting. In contrast, laser + MH-PLGA-IR780 NPs almost completely inhibited the growth of tumors, demonstrating a significant tumor inhibition effect in vivo, which is consistent with the photodynamic cytotoxicity in vitro. Subsequently, the trends in tumor size and weight were measured ex vivo and are shown in Fig. 9C, D. No obvious change in mouse weights between the control and treated groups was observed during the experimental period (Additional file 1: Fig. S8B).

Furthermore, we evaluated the expression of proliferation markers, including PCNA and Ki67, to further validate the inhibitory effects of different treatments on tumor growth. The expression levels of PCNA and Ki67 in tumor specimens measured by IHC were lower in the laser + PLGA-IR780 NPs group than in the control, laser alone, and two single NP groups, while the laser + MH-PLGA-IR780 NPs group showed the lowest expression. Xenograft tumor samples were stained with H&E after mice were sacrificed, as tumor necrosis (karyopyknosis, karyorrhexis, and karyolysis) is a crucial criterion for evaluating the response to various treatments in HOS cells [47]. We found that the specimens from the two PDT groups showed different degrees of necrosis, while specimens from the laser + MH-PLGA-IR780 NPs group exhibited the most severe necrosis (Fig. 9E, Additional file 1: Fig. S8C, D). To evaluate the therapeutic biosafety of PDT in vivo, H&E staining of the main organs (heart, liver, spleen, lung and kidney) was performed at the end of the various treatments, and routine blood and biochemical analysis also showed no significant differences among the various groups (Additional file 1: Fig. S9). In addition, no apparent histopathological abnormalities were observed via H&E staining (Additional file 1: Fig. S10). Taken together, these findings indicated that MH-PLGA-IR780 NPs could enhance PDT performance in a xenograft model with high therapeutic potential and biosafety.

### Ferroptosis-promoted targeted PDT performance

**Fig. 10:**
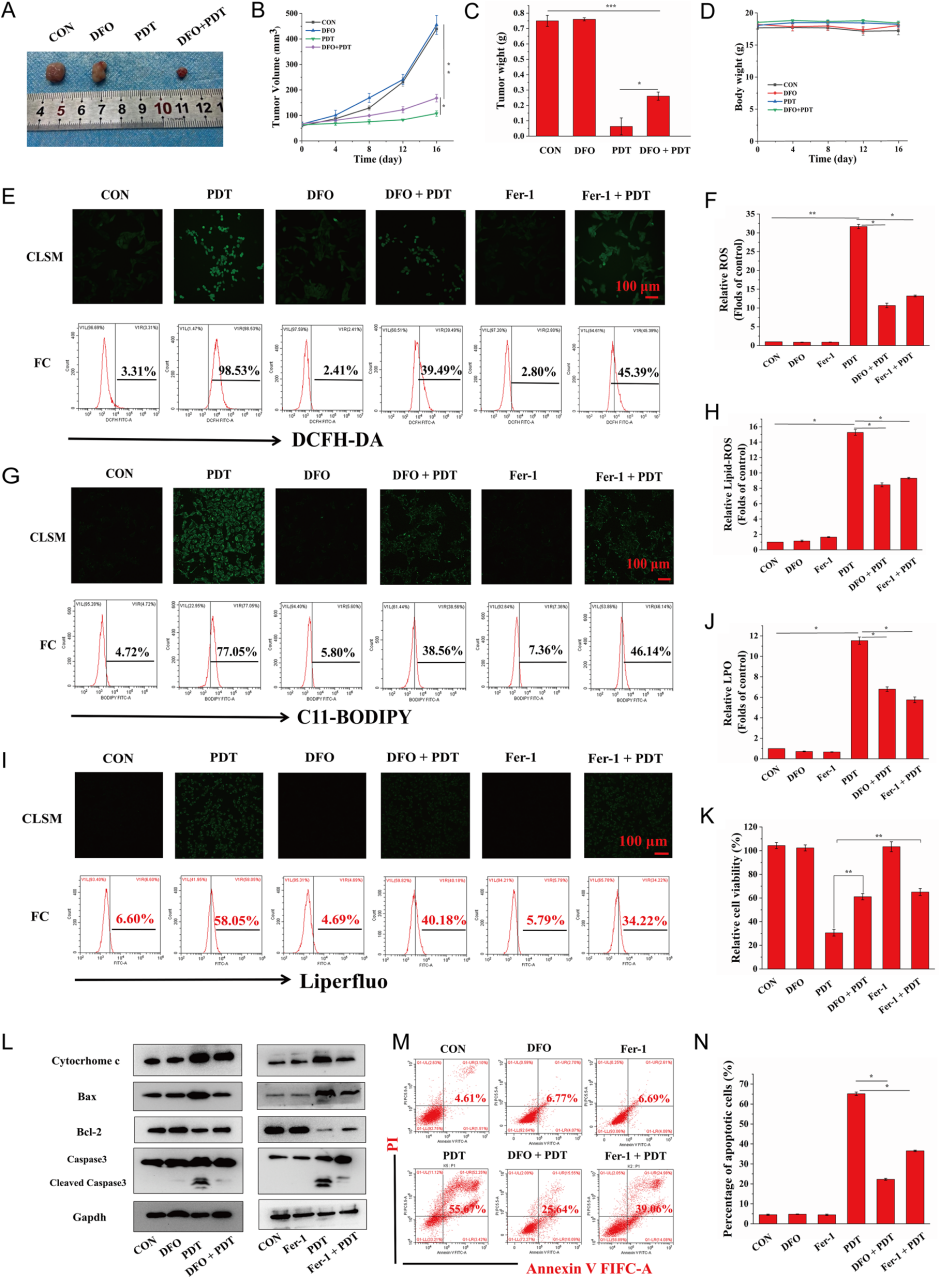
**A** Representative images of tumors collected from mice after various therapies (single DFO dose: 20 mg/kg). **B**–**D** Tumor volume, tumor weight, and mouse weight were measured during the therapeutic period. (The data are presented as the mean ± SD values; n = 5, *p < 0.05, **p < 0.01.) **E**–**J** CLSM and FC analyses of the intracellular ROS, LPO, and lipid-ROS levels by PDT after pretreatments of DFO and Fer-1. (The data are presented as the mean ± SD values; n = 3, *p < 0.05, **p < 0.01.) The scale bars represent 100 µm. **K** The decreased relative cell viability (%) of HOS cells of PDT via preincubation of DFO and Fer-1. (The data are presented as the mean ± SD values; n = 3, **p < 0.01). **L** After ferroptosis scavenging by DFO and Fer-1, the expression levels of cell apoptosis-related proteins induced by PDT were measured by western blot analysis. **M**–**N** The suppression of cell apoptosis percentages (stained with annexin V-FITC/PI) by the inhibition of ferroptosis as determined by FC. The data are presented as the mean ± SD values; n = 3, *p < 0.05.) PDT: laser + MH-PLGA-IR780 NPs

As a new mode of RCD, ferroptosis is desirable to improve the production of free radicals via the Fenton reaction, resulting in an increase in ROS-mediated oxidative damage to cellular constituents and ensuring the high efficiency of PDT. To further determine whether ferroptosis induced by targeted PDT could enhance the original killing characteristics of PDT in vivo, we administered PDT-targeted therapy to HOS xenograft tumor-bearing mice after a combination injection of MH-PLGA-IR780 NPs and DFO, a ferroptosis inhibitor that functions by chelating iron. The tumor size was measured every 4 days over 16 days in the above treatment groups, and it was found that compared with the significant inhibitory effects of the targeted PDT, DFO weakened tumor growth in the xenograft model by inhibiting ferroptosis. Visualization and measurement of the weights of the representative tumors ex vivo showed a similar trend, with no significant alterations in body weight (Fig. 10A–D). These results indicated that activation of ferroptosis allowed the engineered nanoplatform-mediated PDT to promote antitumor effects in vivo.

To investigate the relationship between apoptosis and ferroptosis for PDT in vitro, we sought to clarify whether the inhibition of ferroptosis could protect HOS cells from the photodynamic cytotoxic effects of the targeted PDT. HOS cells were preincubated with DFO for 24 h followed by targeted PDT intervention. Since ferroptosis induction by PDT guided by this nanoplatform was also dependent on tailored lipid peroxidation, HOS cells were also pretreated with another ferroptosis inhibitor, Fer-1, for 24 h before targeted PDT intervention. As shown in Fig. 10E–J, both DFO and Fer-1 reduced PDT performance in terms of intracellular ROS, lipid ROS, and LPOs, as shown by the weaker green FL intensities in CLSM, consistent with the results obtained by FC. Considering that DFO inhibited ferroptosis by depleting intracellular Fe^2+^ and indirectly blocking lipid peroxidation, the content of intracellular Fe^2+^ was detected using FerroOrange, and as shown in Additional file 1: Fig. S11, both CLSM imaging and FC analyses revealed the effective inhibition of intracellular Fe^2+^ during PDT by pretreatment with DFO. In summary, these results indicated that effective blockade of ferroptosis might partly protect HOS cells from the cytotoxic effects of intracellular ROS, Lipid ROS, and LPOs induced by targeted PDT. Additionally, the CCK-8 assay showed that pretreatment with Fer-1 or DFO followed by targeted PDT intervention effectively increased the viability of HOS cells (Fig. 10K). Moreover, to further investigate the relationship and the potential signaling pathways between the apoptosis and ferroptosis induced by PDT guided by this nanoplatform, as shown in Figs. 10L and Additional file 1: Fig. S12, both Fer-1 and DFO attenuated apoptosis by reducing cytochrome c and Bax protein levels, which is consistent with the upregulation of Bcl-2 protein expression, ultimately resulting in a decreased protein level of cleaved caspase-3. Finally, annexin V-FITC/PI double staining analysis showed that HOS cells pretreated with Fer-1 or DFO and then subjected to targeted PDT exhibited a marked decrease in the total apoptosis rate compared with that of cells subjected to targeted PDT without ferroptosis inhibitor pretreatment (Fig. 10M, N). Taken together, these results indicated that the induction of ferroptosis by this theranostic nanoplatform-guided PDT approach could contribute to PDT-mediated apoptosis and inhibit tumor growth in mouse xenografts.

## Discussion

As an emerging treatment characterized by noninvasiveness, high selectivity and few side effects, conventional PDT strategies are exactly focused on superficial cancers. The application of PDT in the clinic is restricted to peripheral and endoscope-accessible regions (such as the skin, neck, and oral cavity) [48, 49]. An effective therapeutic response of deep-seated tumors is hindered by the limited tissue penetration depth of the excitation light and poor in vivo targeting of PSs [50, 51]. In our constructed nanoplatform (MH-PLGA-IR780 NPs), cancer cell membrane-coated NPs had the advantages of high spatial resolution and deep penetration [32]. In addition, with the development of light sources, deep PDT uses two-photon excitation [52], X-ray [53], or NIR-II [54] as light sources to provide better penetration ability to treat tumors lying under deep tissues [55]. We will try to construct multifunctional NPs with deeper PDT penetration in future research. For OS, surgery is the main clinical treatment and will be difficult to replace with future treatment. PDT can be used as a neoadjuvant treatment (NAT) in conjunction with conventional surgery to remove residual tumors in the palliative treatment of larger tumors with nanotechnology and deeper penetrable light sources.

Studies have reported that PLGA-coated PSs can enhance PDT performance in different cancers, such as breast cancer and prostate cancer [25, 38, 56]. However, few studies have been conducted to examine the combination of nanoscale drug-delivery systems and PDT in OS treatment. The lack of targeting to OS cells also limits PDT efficiency. The problem of poor targeting of PSs could be solved by encapsulation in NPs, which could further increase the in vivo circulation times of PSs and the amount of ROS at tumor sites. As a potential method, ligand-receptor binding may solve the problem of poor tumor targeting caused by immune rejection of the mononuclear phagocyte system (MPS) [57], but no specific and effective receptor has been found for the clinical treatment of OS [58]. In the present study, modification of NPs using biofilms such as erythrocyte and macrophage membranes could endow NPs with some of the characteristics of the original cells [59]; for example, coating NPs with red blood cell membranes could prolong their blood circulation time [60]. However, compared with cancer cell membranes, positive tumor targeting cannot be implemented easily due to the lack of targeting molecules on the surface of other types of cell membranes [9, 10].

Mitochondrial dysfunction plays a key role in the regulation of tumor cell death. (i) The mitochondria of tumor cells have a higher Δψm and produce less ATP, which makes the mitochondria of tumor cells more sensitive to mitochondrial targeted drugs [61]. (ii) Mitochondrial dysfunction can cause depolarization of Δψm, promote the release of cytochrome c, and activate the endogenous apoptosis pathway mediated by cleaved caspase-9, cleaved caspase-7, and cleaved caspase-3 [47]. (iii) ROS act as a “double-edged sword” to damage proteins, lipids, and oxidative phosphatases [62], which are produced by PDT and come mainly from mitochondria.

Regarding the function of IR780, some studies have reported that the lipophilic and cationic properties of IR780 could contribute to its preferential intracellular accumulation in the mitochondria of tumor cells [3, 63]. However, based on our preliminary experimental results, the phagocytic capability and tumor/mitochondrial targeting effects of the PLGA-IR780 NPs are not satisfactory for OS cells, a limitation that may be related to tumor heterogeneity and the biocompatibility of the NPs. Subsequently, we constructed a nanoplatform with homologous targeting capacity to successfully target OS cells and enhance the delivery efficiency of IR780, which could act as an excellent PS and a mature imaging probe in the theranostic nanoplatform (MH-PLGA-IR780 NPs).

Induction of a single cell death mode may fail to eradicate tumor cells due to the complex tumor microenvironment and tumor heterogeneity [18]. Fortunately, the increase in intracellular ROS and mitochondrial dysfunction caused by this homologous targeting theranostic nanoplatform-mediated PDT approach resulted in a close relationship between cell apoptosis and ferroptosis. The induction of apoptosis involves two classical pathways: the death receptor pathway (extrinsic pathway) and the mitochondrial pathway (intrinsic pathway) [64]. In the mitochondrial apoptotic pathway, a decrease in Δψm leads to an increase in mitochondrial membrane permeability, which further results in the release of cytochrome c from mitochondria into the cytoplasm. In the cytoplasm, cytochrome c initiates the activation of caspases and eventually induces cell apoptosis. The release of cytochrome c is prevented by the antiapoptotic protein Bcl-2. Furthermore, we observed a decrease in Bcl-2 expression accompanied by an increase in the levels of the proapoptotic proteins Bax, cytochrome c, cleaved caspase-9, cleaved caspase-7, and cleaved caspase-3 by western blot analysis. Due to the distinct differences between ferroptosis and apoptosis, compared with induction of a single cell death mode, simultaneous induction of apoptosis and ferroptosis is expected to overcome the limitations imposed by tumor heterogeneity and improve PDT performance.

To further investigate the promotional or antagonistic role of ferroptosis in mediating apoptosis in HOS cells after treatment with nanoplatform-mediated PDT in vitro and in vivo, we assessed cell viability and apoptosis after blocking ferroptosis with Fer-1 and DFO. Our results showed that after pretreatment with the ferroptosis inhibitors Fer-1 and DFO in HOS cells followed by targeted PDT, the apoptosis rate and cell viability were significantly reduced, and that inhibition of ferroptosis via iron chelation significantly reduced the performance of targeted PDT in suppressing tumor growth compared with that in the group without DFO treatment. These results suggest that ferroptosis plays a key role as a “death switch” in this homologous targeting theranostic nanoplatform-mediated PDT approach.

## Conclusion

In summary, we successfully constructed an emerging theranostic nanoplatform (MH-PLGA-IR780 NPs) with tumor targeting ability accomplished by homologous targeting. Because of its superior FL/PA imaging performance, this nanoplatform is desirable for precise tumor diagnosis and therapy. Combining these targeting NPs with NIR irradiation synergistically induced apoptosis and ferroptosis to improve PDT efficiency in vitro and in vivo. In addition, our results suggest that the underlying mechanisms of apoptosis and ferroptosis are initiated by the mitochondrial apoptotic pathway, excessive accumulation of Fe^2+^, and inactivation of GPX4. Finally, blocking ferroptosis inhibited PDT-induced apoptosis by decreasing intracellular ROS and inhibiting the mitochondrial apoptotic pathway, suggesting that ferroptosis acts as a “death switch” in this homologous targeting-associated theranostic nanoplatform.

## Supplementary Information

**Additional file 1: Fig. S1.** (A) Size distribution of PLGA NPs as measured by DLS. The dynamical change of the size of various NPs (PLGA NPs, PLGA-IR780 NPs, MH-PLGA-IR780 NPs) in the (B) DMEM (10% serum) or (C) PBS with the prolonged time points at 4 °C under 5% CO_2_. (D) The relative absorbance intensity of IR780 was determined via UV–vis spectrum at the wavelength of 798 nm and the standard curve of IR780 was drawn. **Fig. S2.** CLSM images of RAW 264.7 cells after 4 h co-incubation of various NPs (PLGA NPs, PLGA-IR780 NPs and MH-PLGA-IR780 NPs). The scale bars are 25 µm. **Fig. S3.** (A) The standard curve of ATP was measured by luminometer with multimode reader. (B) The relative cell viability (%) of HOS cells of PDT via the pretreatment of various cell death inhibitors (apoptosis inhibitors (z-VAD-FMK, 20 μM), necrosis inhibitors (Nec-1, 20 μM), autophagy inhibitors (Baf-A1, 100 nM), ferroptosis inhibitors (Fer-1, 20 μM) and a general ROS scavenger (NAC, 10 μM)). (The data are presented as the mean ± SD, n = 3, *p < 0.05, **p < 0.01). PDT: laser + MH-PLGA-IR780 NPs. **Fig. S4.** (A–F) Statistical analyses of Cytochrome C, Bax, Bcl-2, cleaved caspase-9, cleaved caspase-7 and cleaved caspase-3 after different treatments. (The data are presented as the mean ± SD, n = 3, *p < 0.05, **p < 0.01). **Fig. S5.** (A–B) The production of Lipid-ROS and LPOs levels in HOS cells (stained with C11-BODIPY and liperfluo) (The data are presented as the mean ± SD, n = 3, *p < 0.05, **p < 0.01). **Fig. S6.** (A–C) Statistical analyses of SLC7A11 and SLC3A2 after different treatments. (The data are presented as the mean ± SD, n = 3, *p < 0.05, ***p < 0.001). **Fig. S7. (**A) FC analysis of Fe^2+^ generation after different treatments. (The data are presented as the mean ± SD, n = 3, **p < 0.01). (B–D) Statistical analyses of NCOA4, FTH and FTL after different treatments. (The data are presented as the mean ± SD, n = 3, *p < 0.05, **p < 0.01, ***p < 0.001). **Fig. S8.** (A) Representative HOS tumor-bearing mice by different treatments before sacrifice. (B) The body-weight of mice were recorded during the therapeutic period of the six group. (C–D) Statistical analyses of the expression of Ki67 and PCNA. (The data are presented as the mean ± SD, n = 5, *p < 0.05). **Fig. S9.** Assay of blood index (routine blood and biochemistry) after different therapies (n = 5). **Fig. S10.** H&E images of major organs after various treatments. The scale bars are 100 µm. **Fig. S11.** (A–B) CLSM images and FC analyses of the intracellular Fe^2+^ with the pretreatment of DFO before PDT treatment. The scale bars are 100 µm. (C) Statistical analyses of the intracellular Fe^2+^ by different treatments. (The data are presented as the mean ± SD, n = 3, *p < 0.05, **p < 0.01). PDT: laser + MH-PLGA-IR780 NPs. **Fig. S12.** (A–H) Statistical analyses of cytochrome C, Bax, Bcl-2, cleaved caspase-3 by pretreatment of DFO and Fer-1 followed PDT treatment. (The data are presented as the mean ± SD, n = 3, *p < 0.05, **p < 0.01, ***p < 0.001). PDT: laser + MH-PLGA-IR780 NPs.

## Data Availability

The datasets used and analyzed during the current study are available from the corresponding author on reasonable request.

## Abbreviations

OS: Osteosarcoma
PDT: Photodynamic therapy
ROS: Reactive oxygen species
PA: Photoacoustic
FL: Fluorescence
Δψm: Mitochondrial membrane potential
LPOs: Lipid peroxides
GPX4: Glutathione peroxidase4
Fer-1: Ferrostatin-1
DFO: Deferoxamine
CLSM: Laser confocal scanning microscopic
FC: Flow cytometry
TEM: Transmission electron microscopy
