# Correction to: Homologous targeting nanoparticles for enhanced PDT against osteosarcoma HOS cells and the related molecular mechanisms

**DOI:** 10.1186/s12951-022-01492-9

**Published:** 2022-06-16

**Authors:** Yang Wang, Liang Zhang, Guosheng Zhao, Yuan Zhang, Fangbiao Zhan, Zhiyu Chen, Tao He, Yang Cao, Lan Hao, Zhigang Wang, Zhengxue Quan, Yunsheng Ou

**Affiliations:** 1 Department of Orthopedic Surgery, The First Affiliated Hospital of Chongqing Medical University, Chongqing, 400016 People’s Republic of China; 2 Department of Ultrasound, The First Affiliated Hospital of Chongqing Medical University, Chongqing, 400016 China; 3 Department of Orthopedic Surgery, The Second Affiliated Hospital of Chongqing Medical University, Chongqing, 400016 People’s Republic of China; 4 Department of Orthopedic Surgery, Children’s Hospital of Chongqing Medical University, Ministry of Education Key Laboratory of Child Development and Disorders, Key Laboratory of Pediatrics in Chongqing, China International Science and Technology Cooperation Base of Child Development and Critical Disorders, Chongqing, 400014 People’s Republic of China; 5 Department of Ultrasound Imaging, Second Affiliated Hospital of Chongqing Medical University, Chongqing, 400014 People’s Republic of China

## Correction to: Journal of Nanobiotechnology (2022) 20:83 https://doi.org/10.1186/s12951-021-01201-y

Following publication of the original article [1], the authors identified an error in Figs. 2 and 7. The corrected Figs. 2, 7 and the figure caption are given in this correction.

**Fig. 2:**
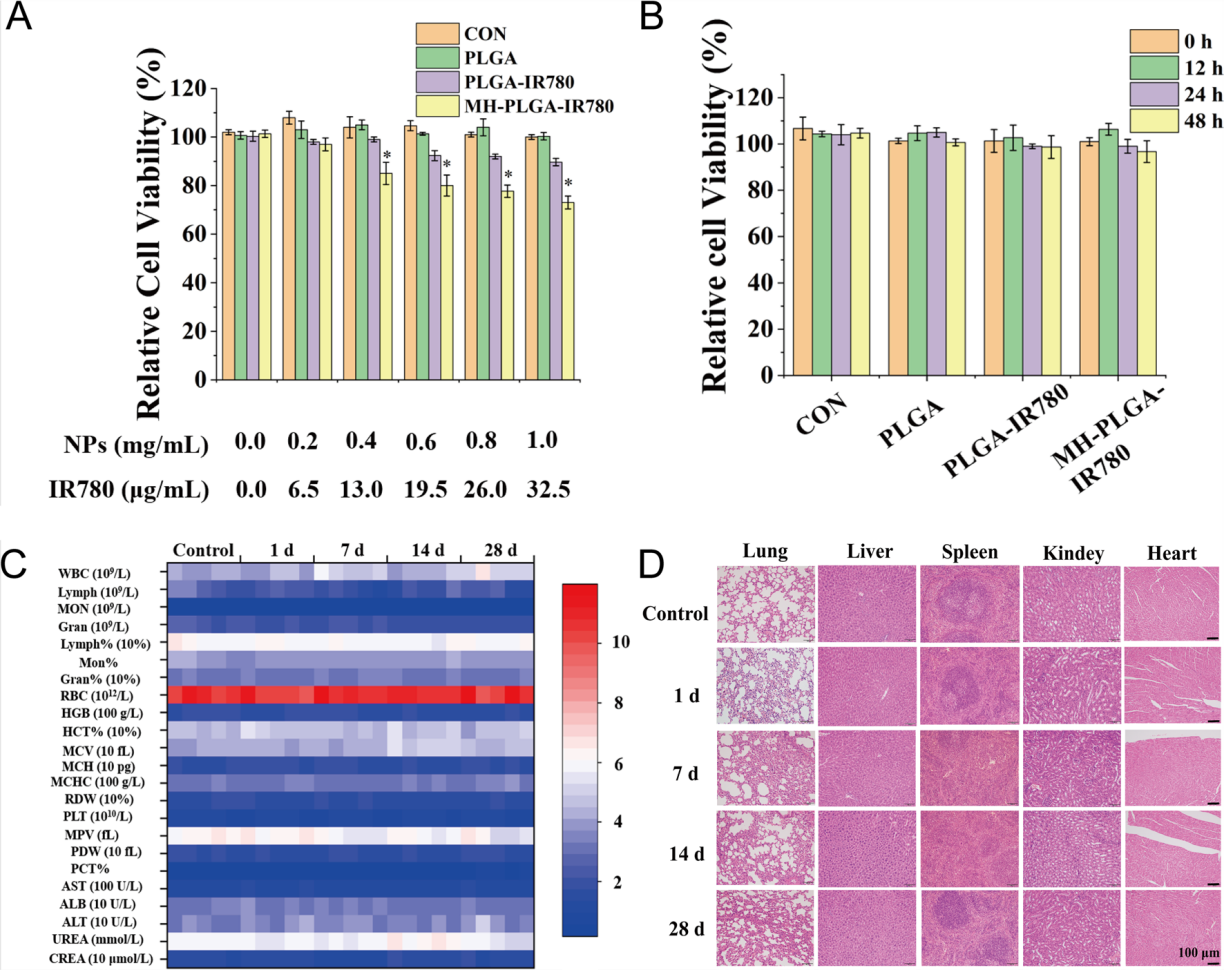
Biosafety of the MH-PLGA-IR780 NPs. **A** Relative viability (%) of HOS cells after coincubation with a wide range of NPs concentrations. **B** Relative viability (%) of HOS cells after coincubation with MH-PLGA-IR780 NPs (PLGA: 0.2 mg/mL) at prolonged time points. (The data are presented as the mean ± SD). **C**, **D** Blood indexes (routine blood and biochemistry) and H&E staining of the main organs (heart, liver, spleen, lung and kidney) of BALB/c nude mice were collected at 0, 1, 7, 14, and 28 days after post injection of MH-PLGA-IR780 NPs (n = 5). The scale bars represent 100 µm

**Fig. 7:**
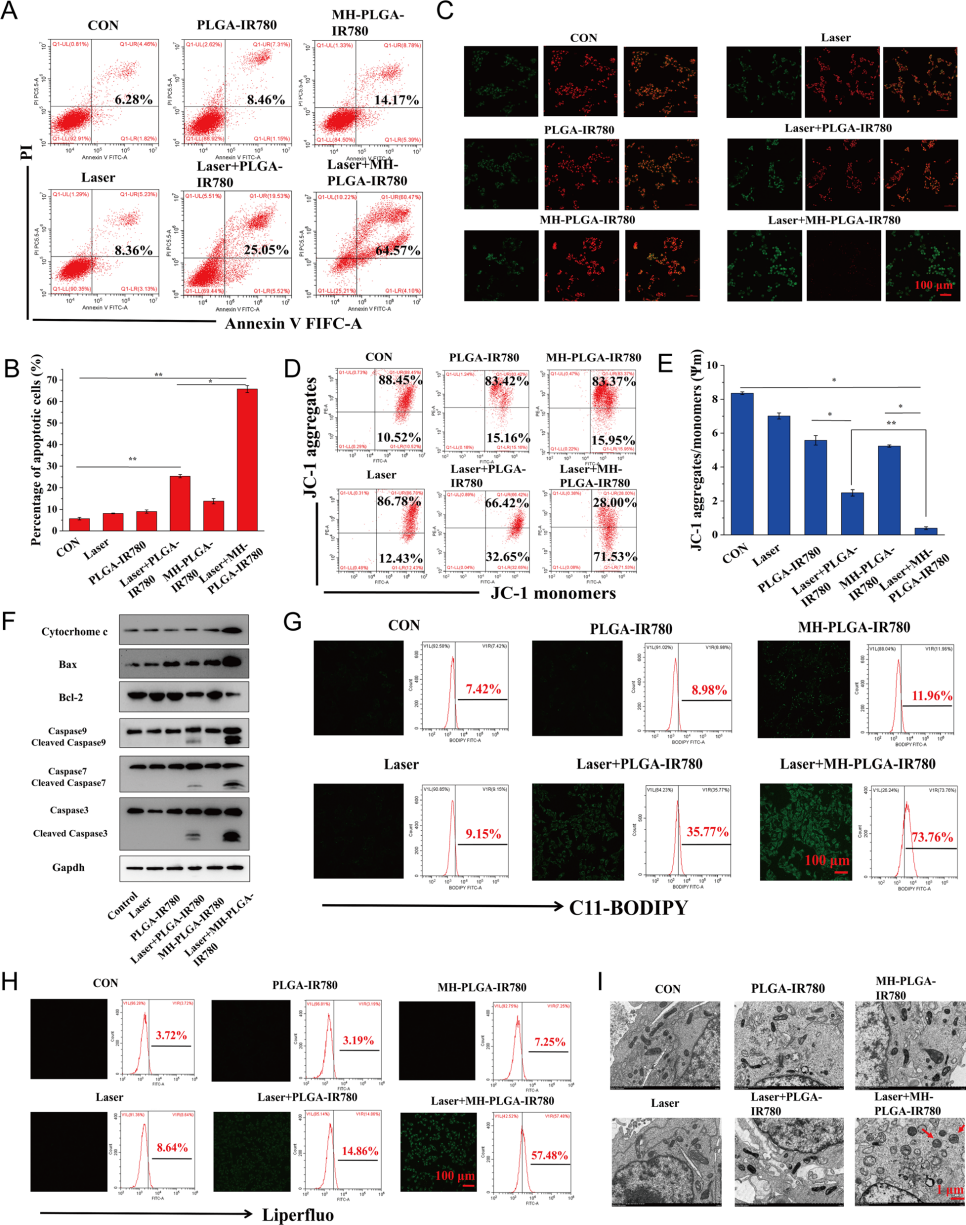
Evaluation of apoptosis and ferroptosis. **A**, **B** Induction of apoptosis in HOS cells (stained with annexin V-FITC/PI) after various treatments by FC analysis. (The data are presented as the mean ± SD values; n = 3, *p < 0.05, **p < 0.01.) **C**–**E** Changes in Δψm in HOS cells stained with JC-1 after various managements, as observed via CLSM and FC. (The data are presented as the mean ± SD values; n = 3, *p < 0.05, **p < 0.01.) The scale bars represent 100 µm. **F** The expression levels of cell apoptosis-related proteins were measured by western blot analysis. **G**, **H** The excessive production of LPO and Lipid ROS in HOS cells after targeted PDT as measured by CLSM and FC. The scale bars represent 100 µm. **I** The morphology of mitochondria after various treatments as observed by TEM. The scale bars represent 1 µm

All the authors apologize for these errors and all the data herein are accurate and reproducible.

The original article has been revised.
