# Supplementary material for: Homologous targeting nanoparticles for enhanced PDT against osteosarcoma HOS cells and the related molecular mechanisms
Source: J Nanobiotechnology. 2022 Feb 17;20:83. doi: 10.1186/s12951-021-01201-y (PMC8851855; doi:10.1186/s12951-021-01201-y)
Supplement: Supplementary file 1 — Additional file 1: Fig. S1. (A) Size distribution of PLGA NPs as measured by DLS. The dynamical change of the size of various NPs (PLGA NPs, PLGA-IR780 NPs, MH-PLGA-IR780 NPs) in the (B) DMEM (10% serum) or (C) PBS with the prolonged time points at 4 °C under 5% CO2. (D) The relative absorbance intensity of IR780 was determined via UV–vis spectrum at the wavelength of 798 nm and the standard curve of IR780 was drawn. Fig. S2. CLSM images of RAW 264.7 cells after 4 h co-incubation of various NPs (PLGA NPs, PLGA-IR780 NPs and MH-PLGA-IR780 NPs). The scale bars are 25 µm. Fig. S3. (A) The standard curve of ATP was measured by luminometer with multimode reader. (B) The relative cell viability (%) of HOS cells of PDT via the pretreatment of various cell death inhibitors (apoptosis inhibitors (z-VAD-FMK, 20 μM), necrosis inhibitors (Nec-1, 20 μM), autophagy inhibitors (Baf-A1, 100 nM), ferroptosis inhibitors (Fer-1, 20 μM) and a general ROS scavenger (NAC, 10 μM)). (The data are presented as the mean ± SD, n = 3, *p < 0.05, **p < 0.01). PDT: laser + MH-PLGA-IR780 NPs. Fig. S4. (A–F) Statistical analyses of Cytochrome C, Bax, Bcl-2, cleaved caspase-9, cleaved caspase-7 and cleaved caspase-3 after different treatments. (The data are presented as the mean ± SD, n = 3, *p < 0.05, **p < 0.01). Fig. S5. (A–B) The production of Lipid-ROS and LPOs levels in HOS cells (stained with C11-BODIPY and liperfluo) (The data are presented as the mean ± SD, n = 3, *p < 0.05, **p < 0.01). Fig. S6. (A–C) Statistical analyses of SLC7A11 and SLC3A2 after different treatments. (The data are presented as the mean ± SD, n = 3, *p < 0.05, ***p < 0.001). Fig. S7. (A) FC analysis of Fe2+ generation after different treatments. (The data are presented as the mean ± SD, n = 3, **p < 0.01). (B–D) Statistical analyses of NCOA4, FTH and FTL after different treatments. (The data are presented as the mean ± SD, n = 3, *p < 0.05, **p < 0.01, ***p < 0.001). Fig. S8. (A) Representative HOS tumor-bearing mice b [file 12951_2021_1201_MOESM1_ESM.docx]

Supporting Information

**Homologous targeting nanoparticles for enhanced PDT against osteosarcoma HOS cells and the related molecular mechanisms**

Yang Wang^1^, Liang Zhang^2^, Guosheng Zhao^3^, Yuan Zhang^4^, Fangbiao Zhan^1^, Zhiyu Chen^1^, Tao He^1^, Yang Cao^5^, Lan Hao^5^, Zhigang Wang^5^, Zhengxue Quan^1^*, Yunsheng Ou^1^*

1. Department of Orthopedic Surgery, The First Affiliated Hospital of Chongqing Medical University, Chongqing 400016, People's Republic of China.

2. Department of Ultrasound, The First Affiliated Hospital of Chongqing Medical University Chongqing 400016, China.

3. Department of Orthopedic Surgery, The Second Affiliated Hospital of Chongqing Medical University, Chongqing 400016, People's Republic of China.

4. Department of Orthopedic Surgery, Children’s Hospital of Chongqing Medical University, Ministry of Education Key Laboratory of Child Development and Disorders, Key Laboratory of Pediatrics in Chongqing, China International Science and Technology Cooperation Base of Child Development and Critical Disorders, Chongqing 400014, People's Republic of China.

5. Department of Ultrasound Imaging, Second Affiliated Hospital of Chongqing Medical University, Chongqing, 400014, People's Republic of China.

*Correspondence to: Professor Yunsheng Ou and Professor Zhengxue Quan.

**
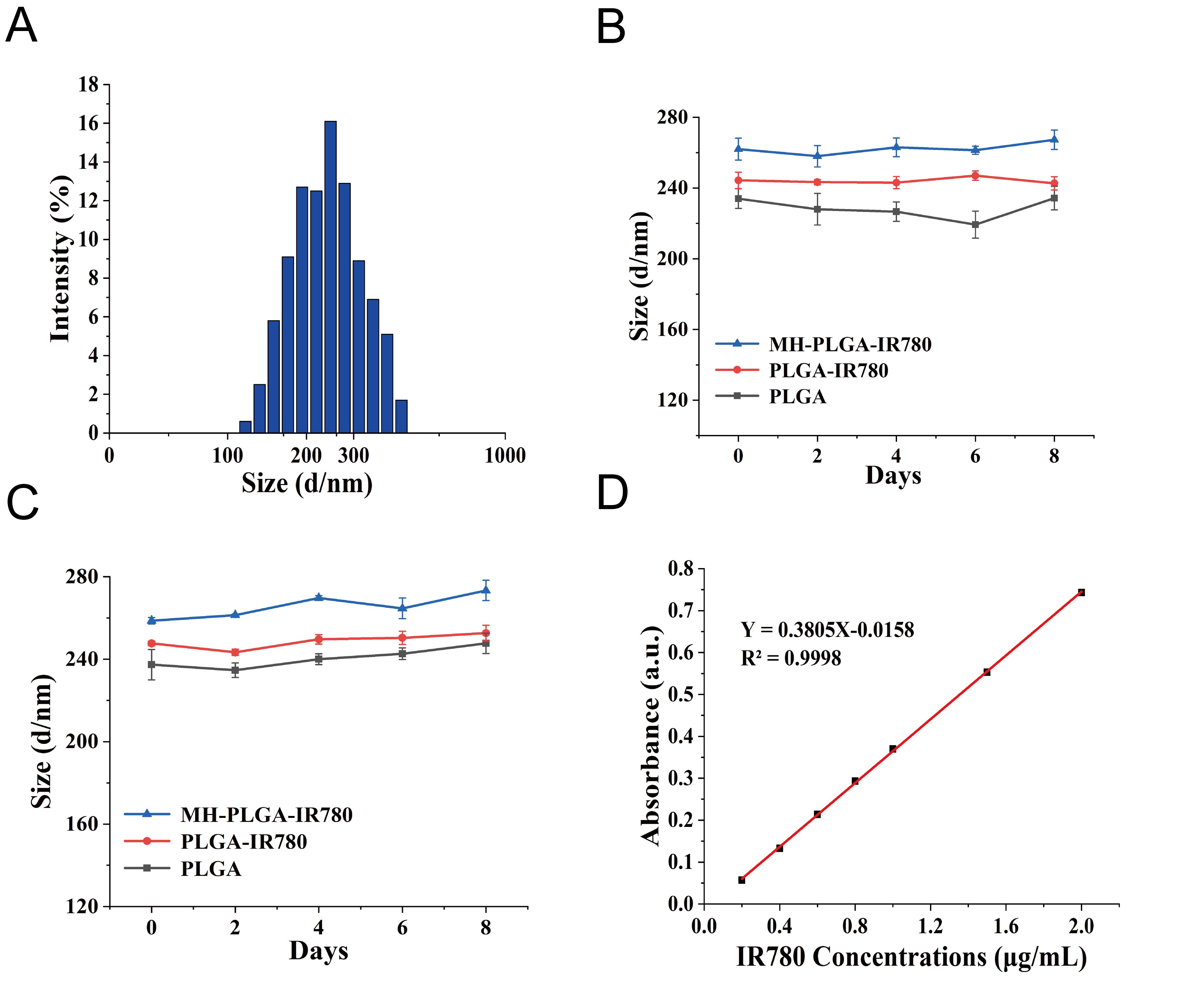
**

**Figure S1.** (A) Size distribution of PLGA NPs as measured by DLS. The dynamical change of the size of various NPs (PLGA NPs, PLGA-IR780 NPs, MH-PLGA-IR780 NPs) in the (B) DMEM (10% serum) or (C) PBS with the prolonged time points at 4°C under 5% CO_2_. (D) The relative absorbance intensity of IR780 was determined via UV–vis spectrum at the wavelength of 798 nm and the standard curve of IR780 was drawn.


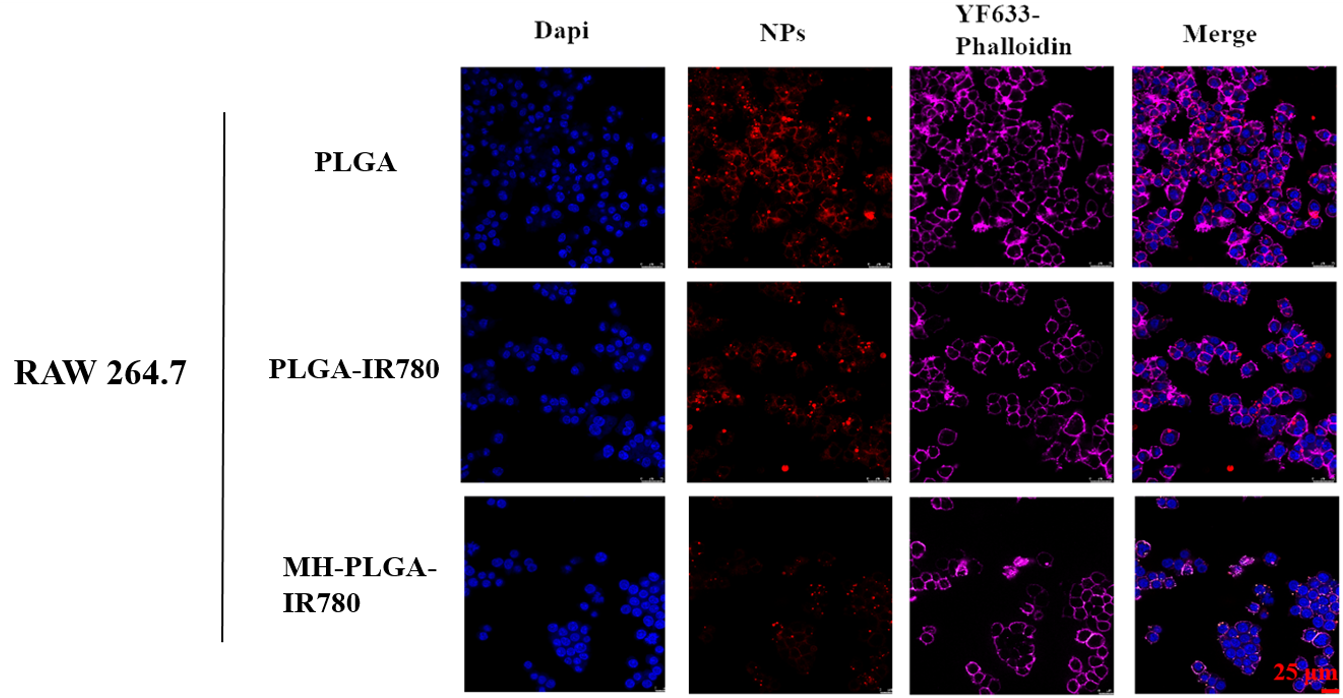


**Figure S2.** CLSM images of RAW 264.7 cells after 4 h co-incubation of various NPs (PLGA NPs, PLGA-IR780 NPs and MH-PLGA-IR780 NPs). The scale bars are 25 µm.

**
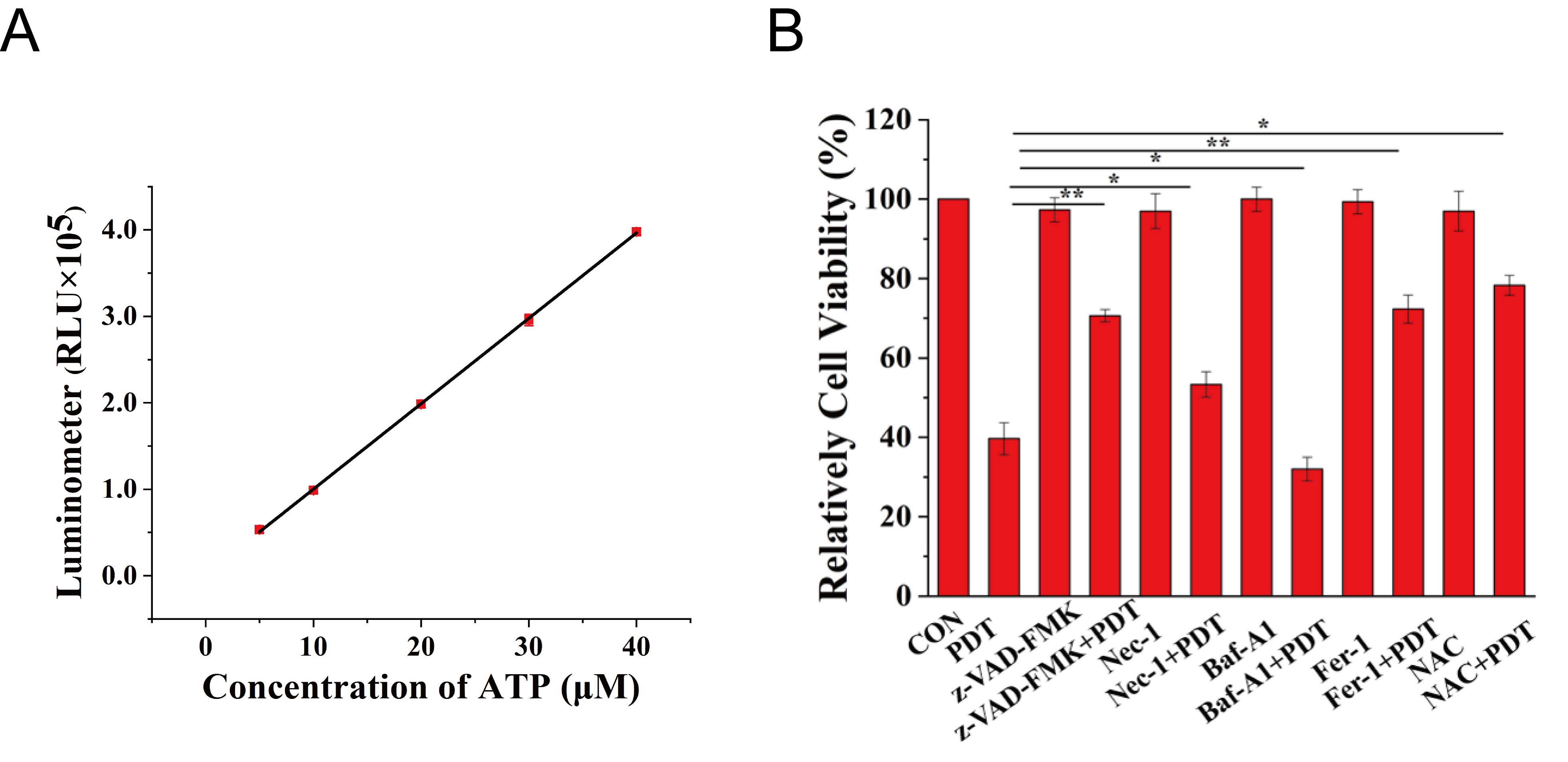
**

**Figure S3.** (A) The standard curve of ATP was measured by luminometer with multimode reader. (B) The relative cell viability (%) of HOS cells of PDT via the pretreatment of various cell death inhibitors (apoptosis inhibitors (z-VAD-FMK, 20 μM), necrosis inhibitors (Nec-1, 20 μM), autophagy inhibitors (Baf-A1, 100nM), ferroptosis inhibitors (Fer-1, 20 μM) and a general ROS scavenger (NAC, 10 μM)). (The data are presented as the mean ± SD, n = 3, *p < 0.05, **p < 0.01). PDT: laser+MH-PLGA-IR780 NPs.


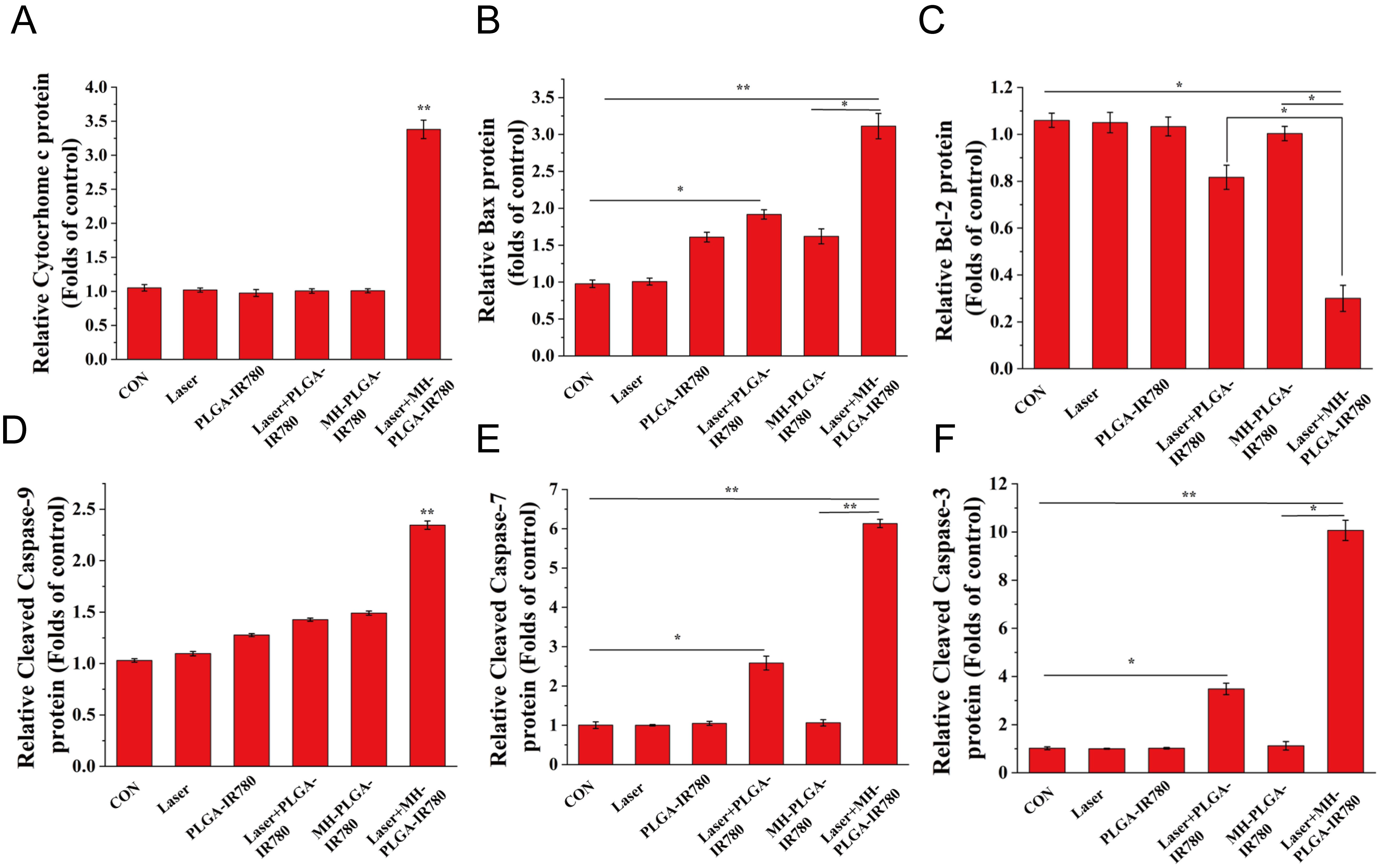


**Figure S4.** (A-F) Statistical analyses of Cytochrome C, Bax, Bcl-2, cleaved caspase-9, cleaved caspase-7 and cleaved caspase-3 after different treatments. (The data are presented as the mean ± SD, n = 3, *p < 0.05, **p < 0.01).


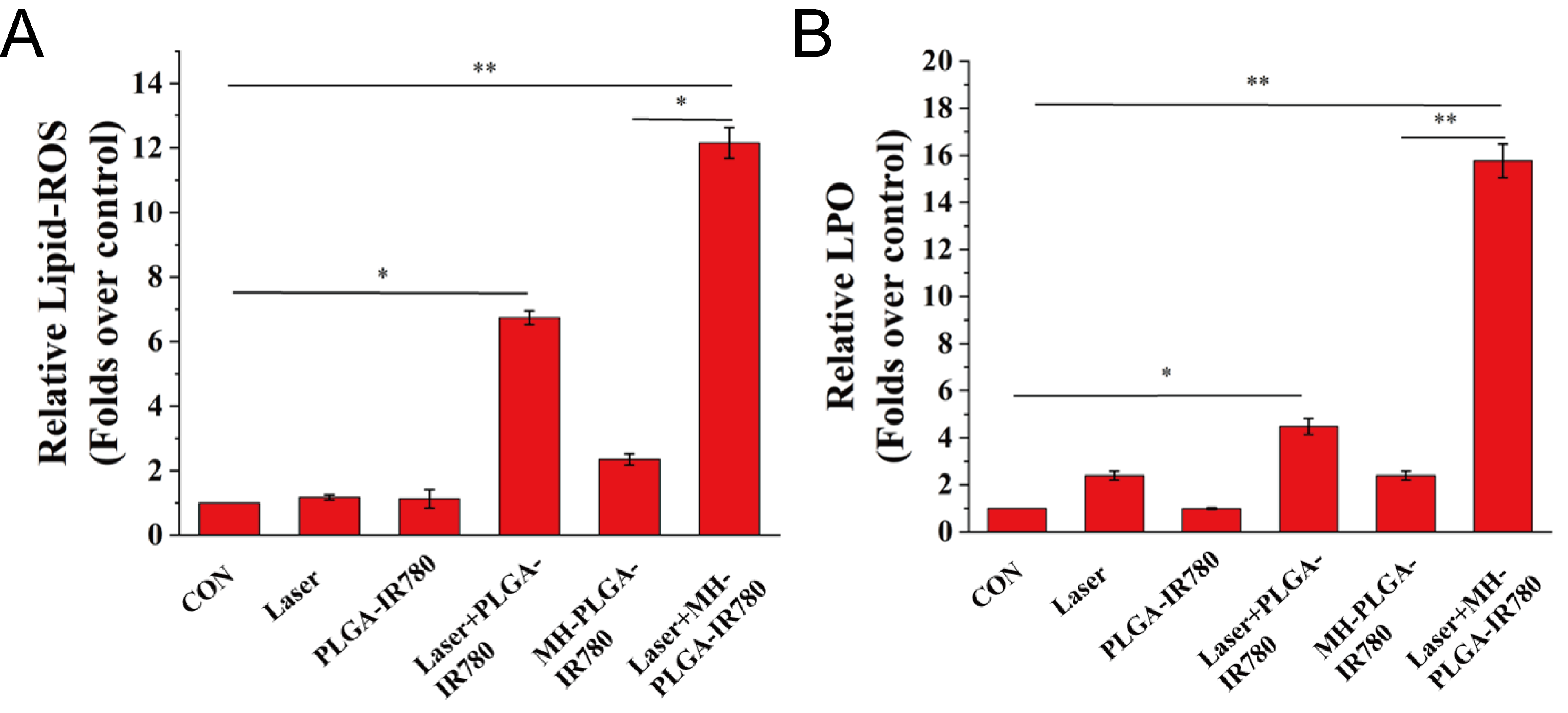


**Figure S5.** (A-B) The production of Lipid-ROS and LPOs levels in HOS cells (stained with C11-BODIPY and liperfluo) (The data are presented as the mean ± SD, n = 3, *p < 0.05, **p < 0.01).


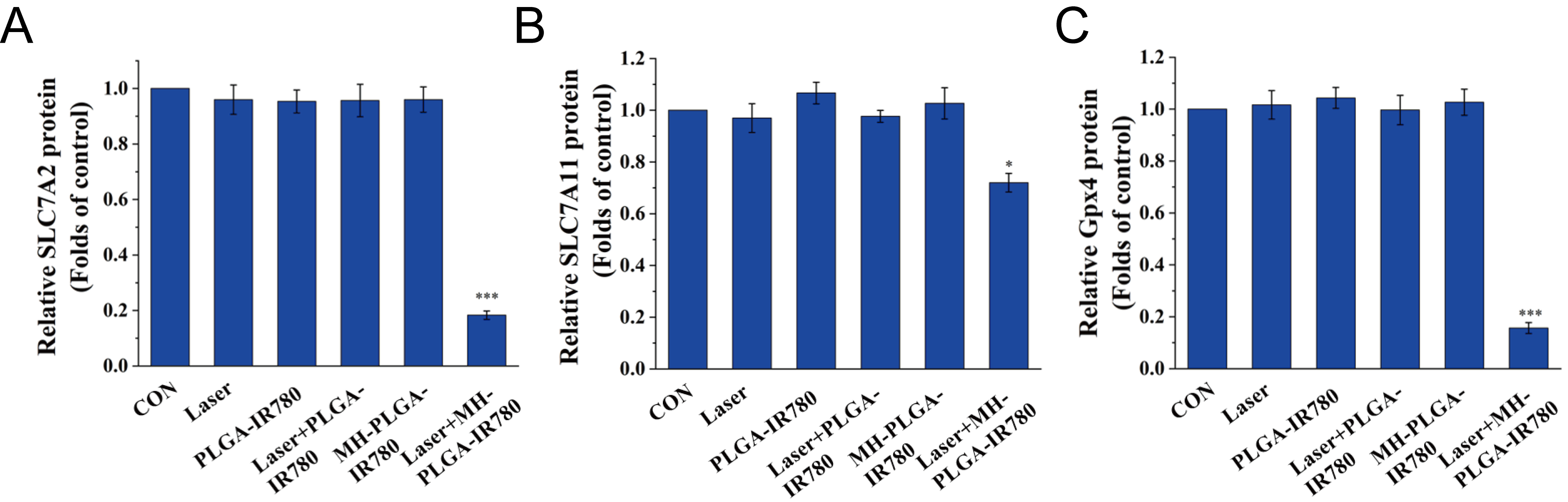


**Figure S6.** (A-C) Statistical analyses of SLC7A11 and SLC3A2 after different treatments. (The data are presented as the mean ± SD, n = 3, *p < 0.05, ***p < 0.001).


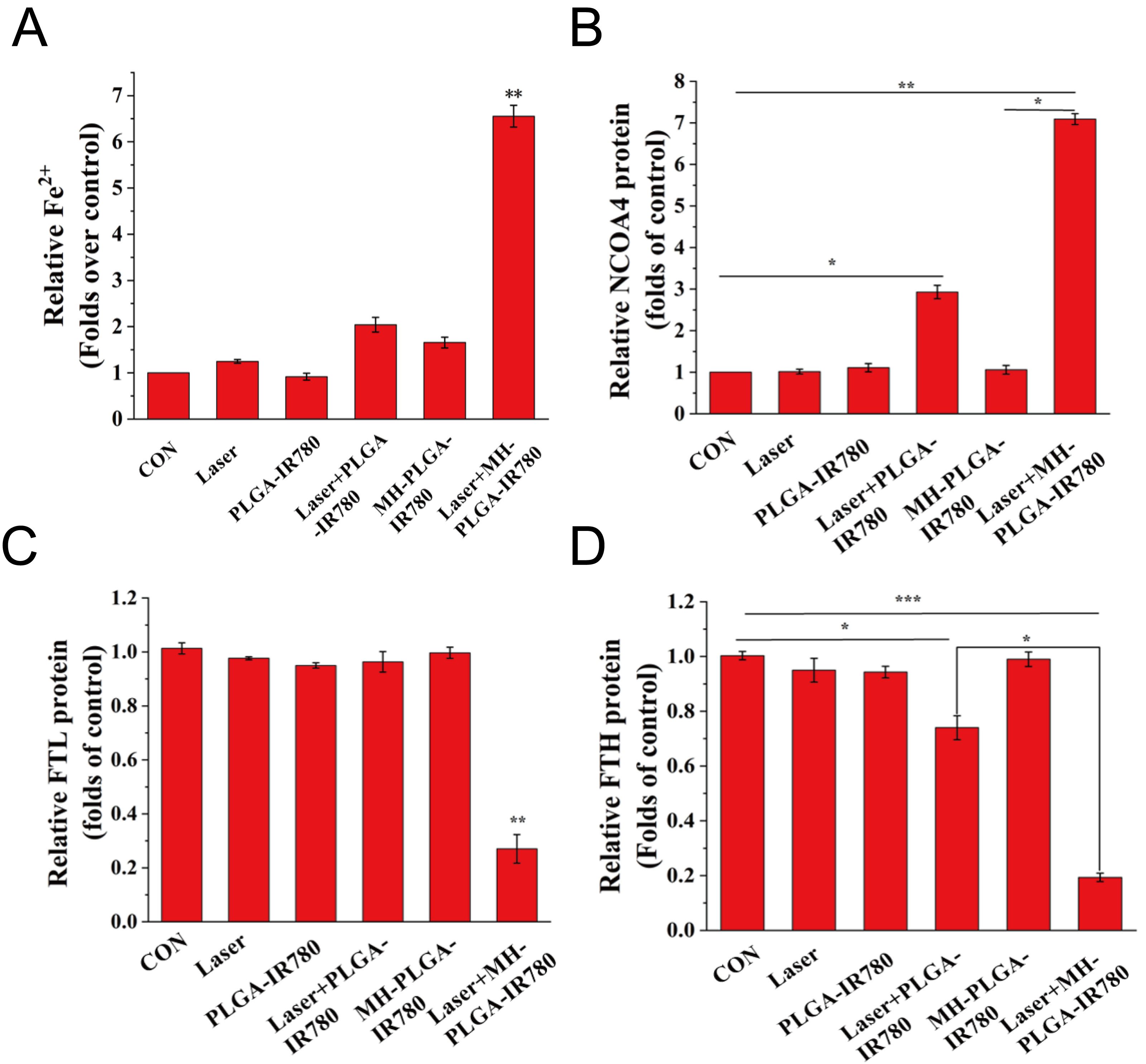


**Figure S7. (**A) FC analysis of Fe^2+^ generation after different treatments. (The data are presented as the mean ± SD, n = 3, **p < 0.01). (B-D) Statistical analyses of NCOA4, FTH and FTL after different treatments. (The data are presented as the mean ± SD, n = 3, *p < 0.05, **p < 0.01, ***p < 0.001).


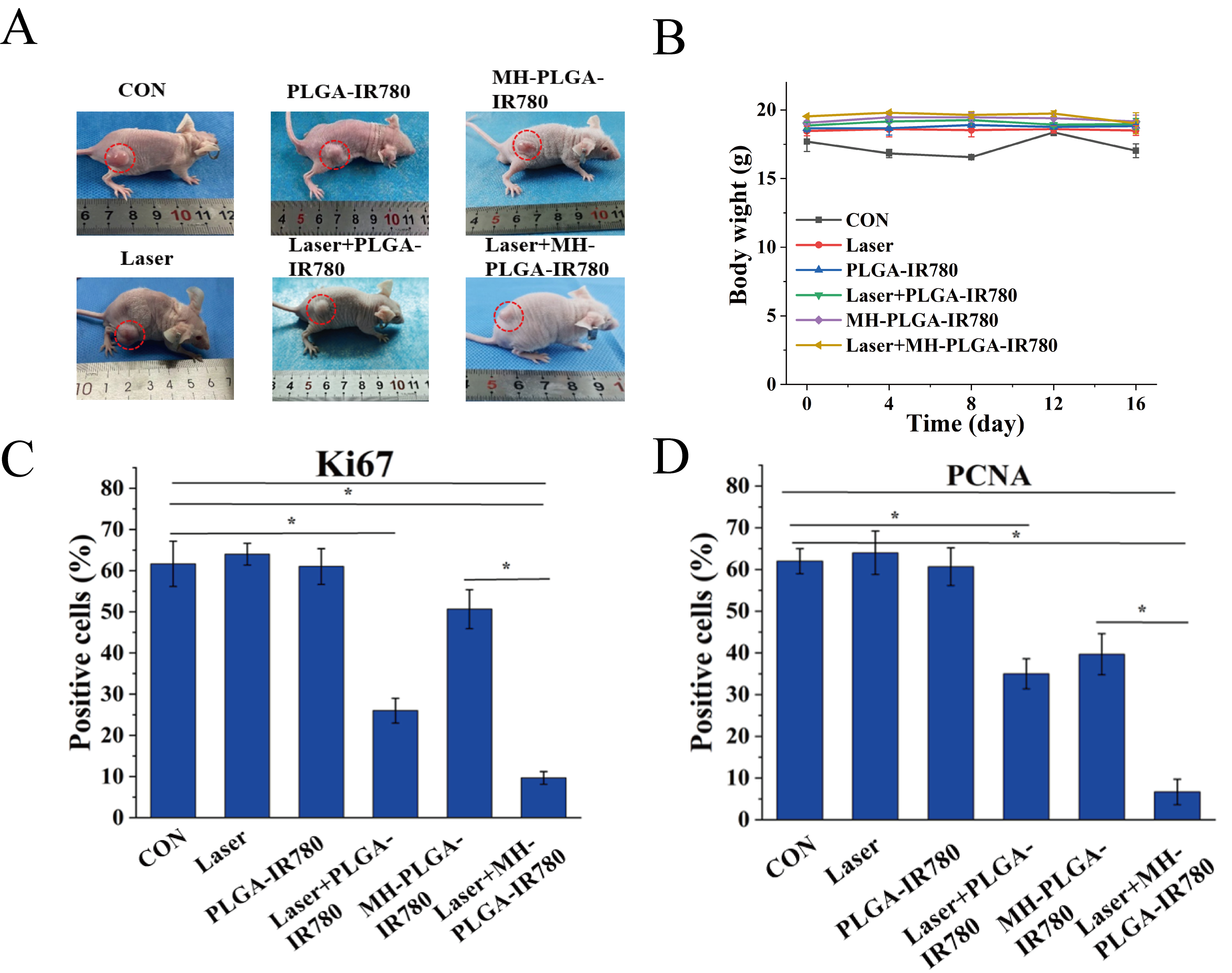


**Figure S8.** (A) Representative HOS tumor-bearing mice by different treatments before sacrifice. (B) The body-weight of mice were recorded during the therapeutic period of the six group. (C-D) Statistical analyses of the expression of Ki67 and PCNA. (The data are presented as the mean ± SD, n = 5, *p < 0.05)

**
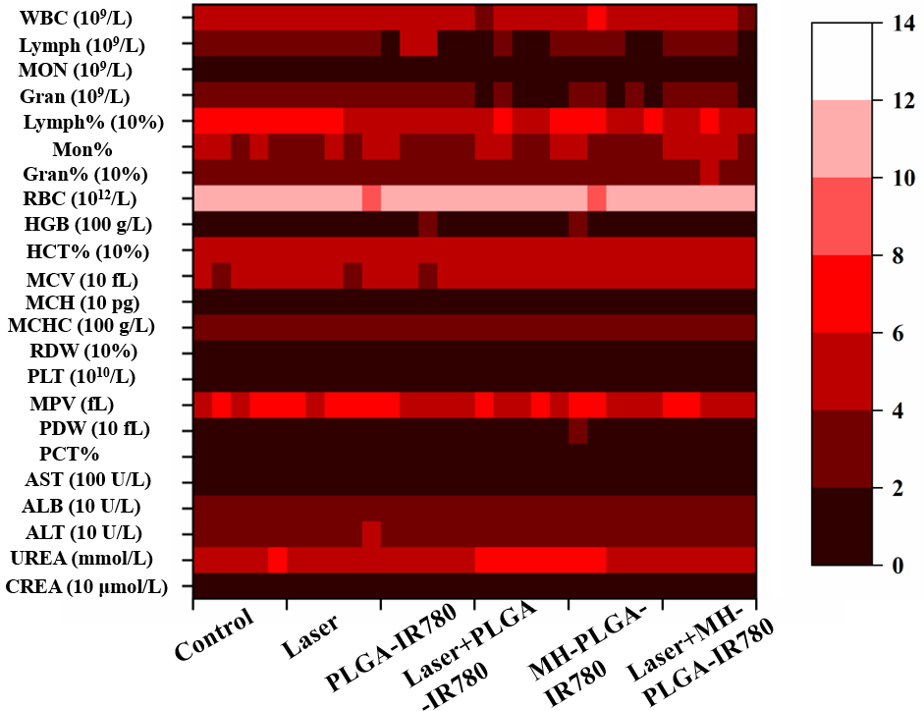
**

**Figure S9.** Assay of blood index (routine blood and biochemistry) after different therapies (n=5).

**
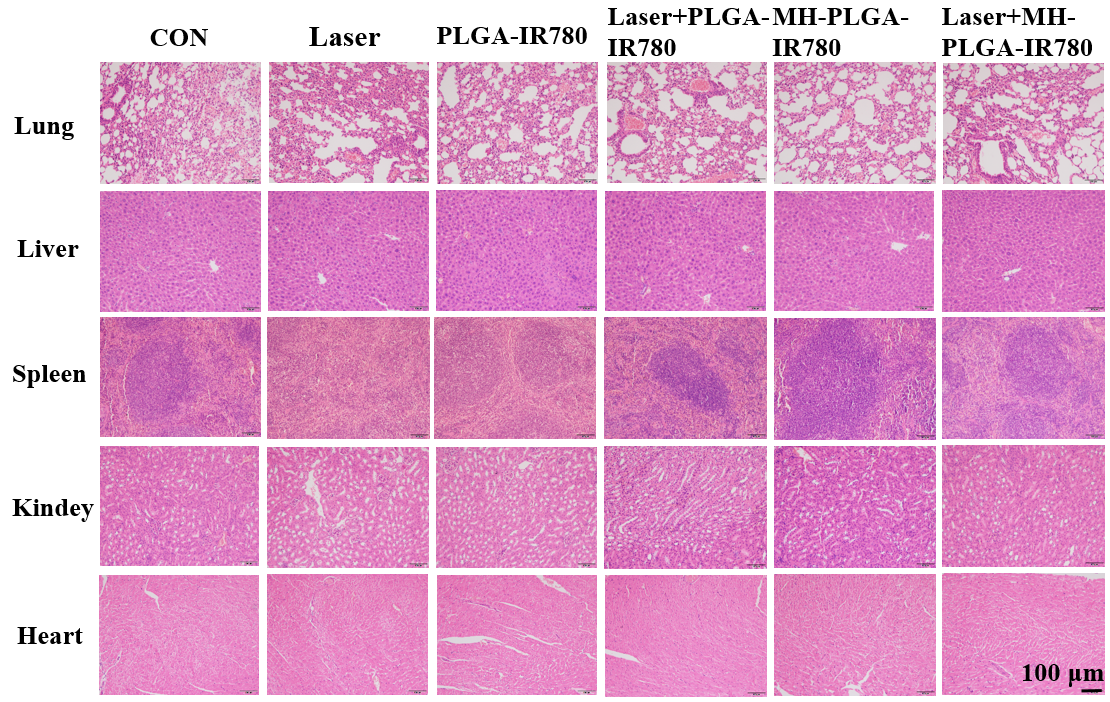
**

**Figure S10.** H&E images of major organs after various treatments. The scale bars are 100 µm.

**
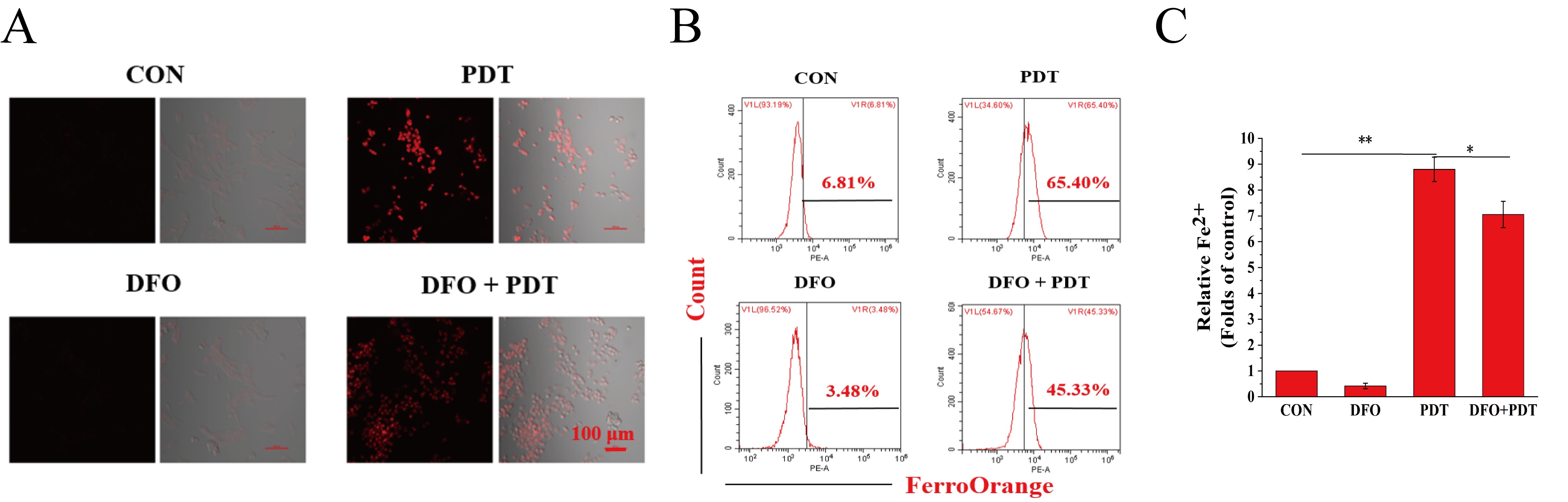
**

**Figure S11.** (A-B) CLSM images and FC analyses of the intracellular Fe^2+^ with the pretreatment of DFO before PDT treatment. The scale bars are 100 µm. (C) Statistical analyses of the intracellular Fe^2+^ by different treatments. (The data are presented as the mean ± SD, n = 3, *p < 0.05, **p < 0.01). PDT: laser+MH-PLGA-IR780 NPs.

**
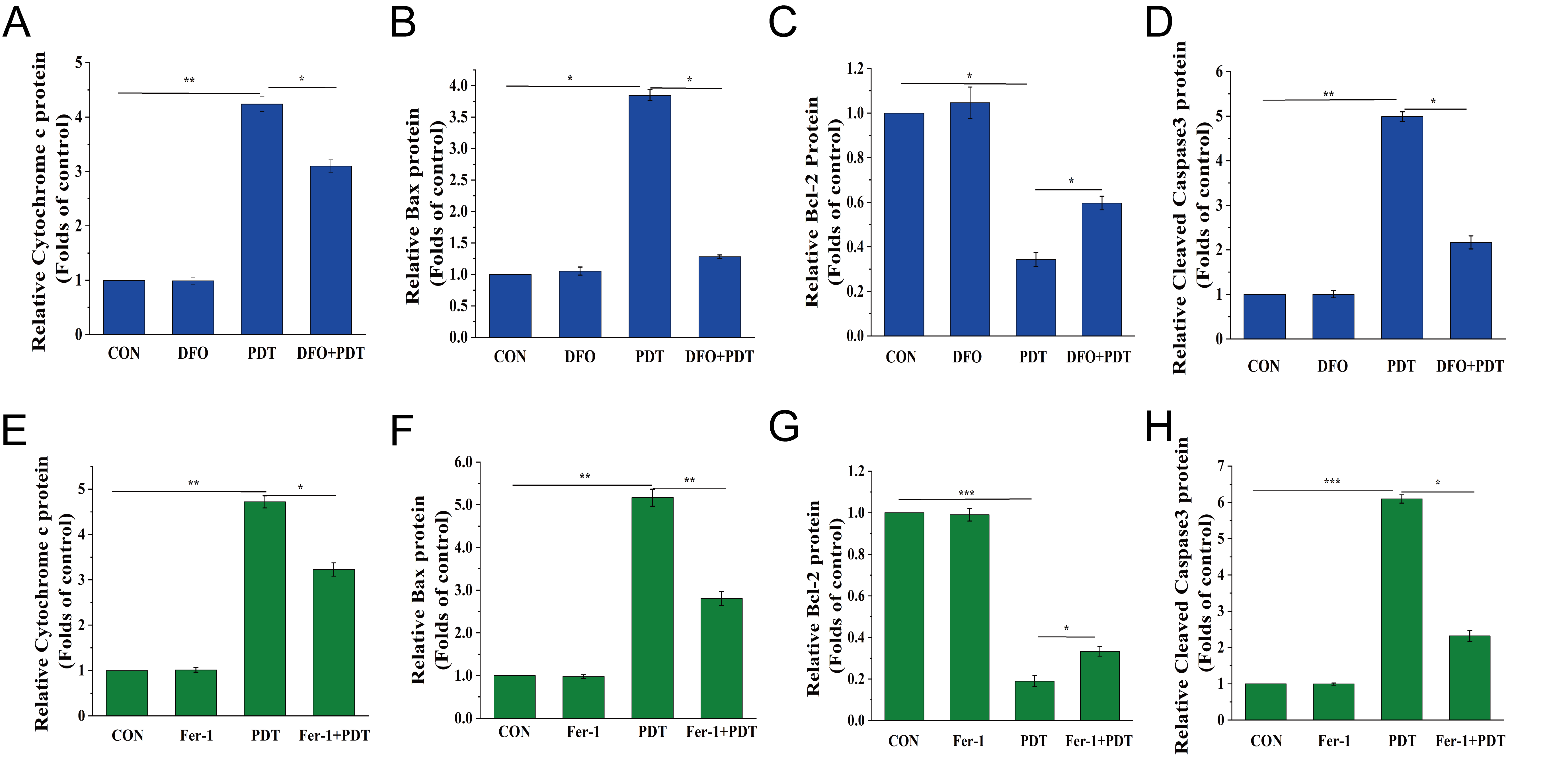
**

**Figure S12.** (A-H) Statistical analyses of cytochrome C, Bax, Bcl-2, cleaved caspase-3 by pretreatment of DFO and Fer-1 followed PDT treatment. (The data are presented as the mean ± SD, n = 3, *p < 0.05, **p < 0.01, ***p < 0.001). PDT: laser+MH-PLGA-IR780 NPs.
